# Type I and II Interferon Signalling Characterizes the Transcriptional Landscape of Sweet Syndrome

**DOI:** 10.1111/exd.70323

**Published:** 2026-07-06

**Authors:** Laura Calabrese, Chiara Moltrasio, Maurizio Romagnuolo, Pia‐Charlotte Stadler, Zeno Fiocco, Matthias Neulinger‐Muñoz, Rui Aoki, Martina D'Onghia, Pietro Rubegni, Katrin Kerl, Takashi K. Satoh, Angelo V. Marzano, Lars E. French

**Affiliations:** ^1^ Dermatology Unit, Department of Medical, Surgical and Neurosciences Siena University Hospital Siena Italy; ^2^ Institute of Dermatology Catholic University of the Sacred Heart Rome Italy; ^3^ Department of Dermatology and Allergy University Hospital LMU Munich Germany; ^4^ Dermatology Unit Fondazione IRCCS Ca' Granda Ospedale Maggiore Policlinico Milan Italy; ^5^ Department of Dermatology Universitätsspital Zürich Zürich Switzerland; ^6^ Dr Phillip Frost Department of Dermatology and Cutaneous Surgery Miller School of Medicine, University of Miami Miami Florida USA; ^7^ Department of Pathophysiology and Transplantation Università Degli Studi di Milano Milan Italy

**Keywords:** autoinflammatory diseases, immunity, inflammatory skin diseases, innate

## Abstract

Sweet syndrome (SS) is an autoinflammatory neutrophilic dermatosis characterized by abrupt‐onset inflammatory skin lesions and systemic symptoms, yet its molecular pathogenesis remains incompletely defined. To delineate disease‐specific inflammatory programmes, we performed NanoString‐based transcriptomic analysis of SS skin lesions and compared them with healthy control skin and pyoderma gangrenosum, a related neutrophilic dermatosis. SS exhibited a distinct inflammatory transcriptional signature marked by robust upregulation of type I and II interferon–stimulated genes, including *CXCL9, CXCL10, GBP1, GBP5, IFIT2* and *IRF7*, distinguishing SS from both control groups. Cell type deconvolution analysis revealed enrichment of dendritic cells, consistent with a prominent type I interferon‐driven immune response. In parallel, SS lesions demonstrated altered immunoproteasome gene expression, with upregulation of immunoproteasome subunits *PSMB8, PSMB9* and *PSMB10* and downregulation of the constitutive subunit *PSMB7* suggesting functional remodelling of proteasomal activity. Together, these findings support a model in which dendritic cell‐driven interferon signalling promotes immunoproteasome remodelling and sustains neutrophilic inflammation in Sweet. This study identifies a prominent interferon signalling as a defining molecular feature of SS and highlights potential therapeutic opportunities within the interferon–JAK/STAT and proteasome pathways.

**Abbreviations**
CANDLE/PRAAS: Chronic atypical neutrophilic dermatosis with lipodystrophy and elevated temperature syndrome/proteasome‐associated autoinflammatory syndromes
CCL: C‐C motif chemokine ligand
CXCL: CXC motif chemokine ligand
DEG: differentially expressed gene
GBP1: guanylate‐binding protein 1
GBP5: guanylate binding protein 5
G‐CSF: granulocyte colony‐stimulating factor
GZMB: granzyme B
HCs: healthy controls
IFIT2: interferon induced protein with tetratricopeptide repeats 2
IFN: interferon
IL: interleukin
IRF7: interferon regulatory factor 7
ITGAX: integrin subunit alpha X
JAK/STAT: janus kinase/signal transducer and activator of transcription
LILRB2: leukocyte immunoglobulin like receptor B2
Log_2_FC: log_2_ fold change
MDNCF: monocyte‐derived neutrophil chemotactic factor
MHC: major histocompatibility complex
PCA: principal component analysis
pDCs: plasmacytoid dendritic cells
PG: pyoderma gangrenosum
PSMB: proteasome 20S subunit beta
SS: Sweet syndrome
Th: T helper
TLR: toll‐like receptor
TNF: tumour necrosis factor
UPS: ubiquitin‐proteasome system
VEXAS: vacuoles, E1 enzyme, X‐linked, autoinflammatory, somatic

## 1 Introduction

Sweet syndrome (SS), originally described as acute febrile neutrophilic dermatosis, is a rare, noninfectious inflammatory skin disease characterized by the abrupt onset of painful erythematous‐oedematous plaque‐type lesions, and more rarely pustular, blistering and targetoid manifestations, often accompanied by systemic inflammation, including fever and neutrophilia [1, 2, 3, 4]. Although frequently idiopathic, SS may occur in association with infections, autoimmune or autoinflammatory disorders, malignancies, immunodeficiency states, or drug exposure, and is currently classified into classical, malignancy‐associated, and drug‐induced subtypes [5, 6, 7, 8]. An expanding spectrum of clinical and histopathological variants further highlights the biological heterogeneity of the disease [2, 9, 10, 11].

Despite improved clinical recognition, the molecular mechanisms that define SS and distinguish it from other neutrophilic dermatoses remain incompletely understood. In particular, SS shares substantial clinical, histological and therapeutic overlap with pyoderma gangrenosum (PG), yet the molecular basis underlying their divergence has not been systematically defined. Current pathogenic models emphasize dysregulated innate immunity, with a central role for IL‐1–dependent inflammatory pathways and neutrophil recruitment [12, 13]. Contributions from adaptive immune responses, including Th1‐ and IL‐17‐associated pathways, have also been proposed, although available data remain heterogeneous [14].

Importantly, existing mechanistic insights into SS are largely derived from targeted cytokine analyses or immunohistochemical studies focused on predefined pathways [15, 16, 17, 18]. While informative, these approaches are inherently limited in their ability to capture broader transcriptional programmes, upstream regulatory networks, and disease‐defining immune signatures. A comparative, unbiased transcriptomic strategy is therefore needed to delineate molecular features specific to SS and to distinguish them from related neutrophilic dermatoses.

Here, we hypothesized that SS exhibits a distinct transcriptional landscape that differentiates it from both healthy skin and PG, reflecting disease‐specific inflammatory and immune pathways beyond established IL‐1–centric models. To test this hypothesis, we performed NanoString‐based gene expression analysis of SS skin lesions and compared them with healthy controls and PG, aiming to identify defining cytokine signatures, immune cell populations, and signalling pathways with potential mechanistic and therapeutic relevance.

## 2 Materials and Methods

### 2.1 Patients and Tissue Selection

This study was approved by the Medical Ethics Committee of Ludwig‐Maximilians‐Universität in Munich, Germany (Project No. 22‐0342). Formalin‐fixed, paraffin‐embedded (FFPE) skin samples were obtained from 11 unrelated patients with SS and 10 patients with PG who were recruited and followed at the Dermatology Departments of Fondazione IRCCS Ca′ Granda Ospedale Maggiore Policlinico (Milan, Italy) and at Ludwig‐Maximilians‐Universität (Munich, Germany) between January 2007 and January 2021. Sixteen healthy control skin samples were included as non‐inflamed comparators. Written informed consent was obtained from all participants. Demographic and clinical characteristics of SS patients are summarized in Table S3.

The diagnosis of SS and PG was established based on clinical, laboratory and histopathological criteria and confirmed by an experienced dermatopathologist. SS diagnosis was made according to the modified criteria proposed by von den Driesch [19]. Major criteria included abrupt onset of painful cutaneous lesions and histopathological evidence of a dense neutrophilic infiltrate without leukocytoclastic vasculitis. Minor criteria included fever, laboratory abnormalities, rapid response to systemic corticosteroids, and an association with an underlying inflammatory or infectious condition. A diagnosis of SS required fulfilment of both major criteria and at least two minor criteria. PG cases were diagnosed clinically and histologically in accordance with PARACELSUS Score [20]. Healthy control skin samples were obtained from patients undergoing sentinel lymph node excision for cutaneous melanoma.

### 2.2 NanoString Gene Expression Analysis

RNA was extracted from FFPE samples using the RecoverAll Total Nucleic Acid Isolation Kit for FFPE (Thermo Fisher Scientific) according to the manufacturer's instructions. RNA quantity and quality were assessed using a NanoDrop One/OneC spectrophotometer (Thermo Fisher Scientific). Gene expression analysis was performed using NanoString nCounter technology with a custom panel comprising 594 immune‐related genes, 15 internal reference genes and 30 user‐selected targets (Panel Plus). Data acquisition was conducted using the nCounter SPRINT Profiler and raw counts were generated using nSolver Analysis Software (Nanostring technologies).

Downstream analyses were performed in R (version 4.3.1). Background correction was performed by applying a threshold defined as two standard deviations above the mean of negative control probes. Data were normalized using the advanced normalization method with housekeeping genes selected by the geNorm algorithm and subsequently log_2_‐transformed. Differential expression analysis was performed using a generalized linear model framework, with multiple testing correction by the Benjamini‐Hochberg method and a false discovery rate (FDR) threshold of 0.05.

Cell type profiling was conducted using predefined immune cell gene signatures implemented in the nCounter Advanced Analysis 2.0 workflow, with cell scores calculated as the arithmetic mean of log2‐transformed expression values of marker genes. Statistical significance was assessed using unpaired *t*‐tests with Benjamini‐Hochberg correction for multiple testing (FDR 5%). *p* < 0.05, *p < 0.01, ***p < 0.001. Pathway activity scores were computed using principal component (PC1) representing pathway‐level expression. Heatmaps were generated using hierarchical clustering based on Pearson correlation distances, with *z*‐score‐transformed expression values for visualization.

## 3 Results

### 3.1 Sweet Syndrome Exhibits a Distinct Transcriptional Signature Relative to Healthy Skin and Pyoderma Gangrenosum

**FIGURE 1:**
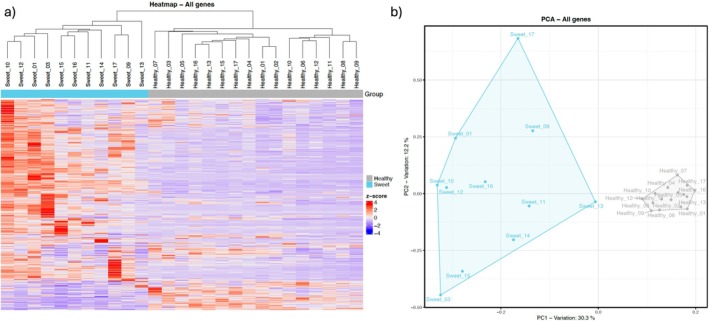
Sweet syndrome exhibits a distinct transcriptional profile compared with healthy skin. (a) Unsupervised hierarchical of NanoString gene expression data from skin samples of patients with Sweet syndrome (SS) and healthy controls (HCs), visualized as a heatmap, demonstrates clear segregation of SS and HC samples. (b) The principal component analysis (PCA) of the same dataset confirms distinct clustering of SS and HC samples, indicating a disease‐associated transcriptional signature in SS.

To define global transcriptional relationships among sample groups, we first performed unsupervised hierarchical clustering using all genes profiled by the NanoString custom panel. SS samples segregated into a distinct cluster clearly separated from healthy controls (HCs), indicating a consistent disease‐associated transcriptional programme (Figure 1a). Principal component analysis (PCA) independently confirmed this separation, with SS and HC samples occupying nonoverlapping regions of transcriptional space. When SS samples were analysed together with PG, clustering revealed partial overlap, but overall segregation between the two diseases, suggesting inflammatory features alongside disease‐specific transcriptional differences (Figure 1b). PCA, similarly, demonstrated partial convergence with preserved separation, supporting molecular relatedness between SS and PG while highlighting distinct underlying programmes. A combined analysis including SS, PG and HC simultaneously is provided in Figure S1.

### 3.2 Interferon–Related Genes Are Selectively Upregulated in Sweet Syndrome

Differential expression analysis comparing SS with HCs identified a highly inflammatory transcriptional profile in SS (Table S1). Among the most strongly upregulated genes were the interferon‐inducible chemokines *CXCL10* and *CXCL9* (Log2FC = 7.62 and 6.98, respectively; adjusted *p* < 0.001), together with *IL8* (Log2FC = 6.39; *p* < 0.001), *GBP5* (Log2FC = 6.59; *p* < 0.001), *IL1B* (Log2FC = 5.52;*p* < 0.001) and *GZMB* (Log2FC = 4.56; *p* < 0.001). SS lesions also exhibited marked upregulation of innate immune sensors, including the pattern recognition receptors *TLR1* (Log2FC = 2.44; *p* = 4.90 × 10^−15^) and *TLR2* (Log2FC = 3.87; *p* = 5.86 × 10^−13^), as well as myeloid‐associated markers such as the adhesion molecule *ITGAX* (CD11c; Log2FC = 5.08; *p* = 1.33 × 10^−12^) and (Log2FC = 4.10; *p* = 5.59 × 10^−14^).

Direct comparison of SS and PG revealed a subset of genes selectively enriched in SS, indicating disease‐specific transcriptional features (Table S2). Notably, multiple interferon‐related genes were significantly upregulated in SS relative to PG, including the gene encoding the protein *GBP1* (Log2FC = 1.93 vs. PG; *p* = 0.0499), *IFIT2* (Log_2_FC = 2.82; *p* = 0.0289), and *IRF7* (Log2FC = 1.64; *p* = 0.0360), which is known to be interferon‐inducible and a trigger of inflammasome activation in response to pathogens [21]. Additional genes enriched in SS compared with PG included *CCL8* (Log2FC = 2.98; *p* = 0.0454) and *GZMB* (Log2FC = 1.16; *p* = 0.0499), further supporting a disease‐specific inflammatory signature.

### 3.3 Immune Cell Deconvolution Reveals Distinct Cellular Enrichment Patterns in Sweet Syndrome

**FIGURE 2:**
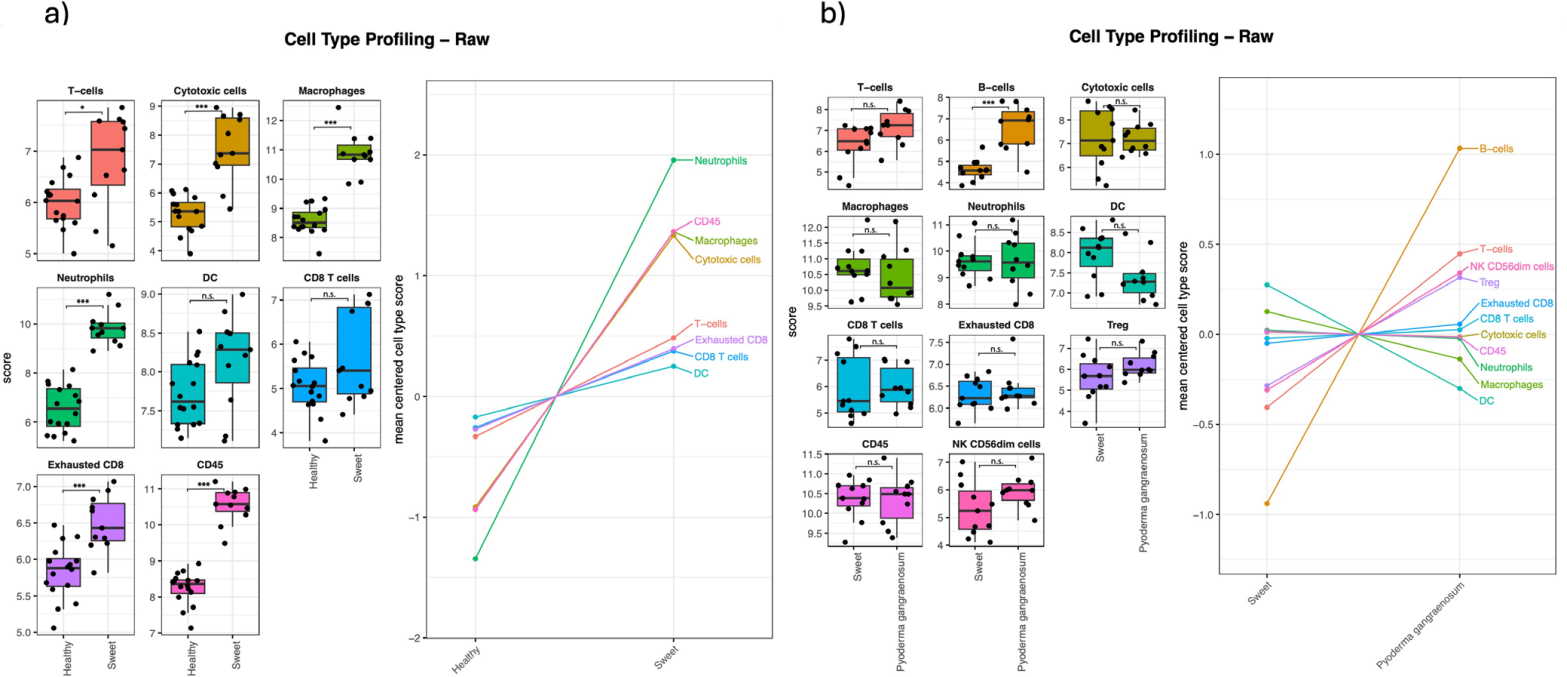
Immune cell deconvolution identifies distinct cellular enrichment patterns in Sweet syndrome and pyoderma gangrenosum. (a) Cell type deconvolution analysis comparing to SS and HC samples reveals marked enrichment of neutrophils in SS, followed by macrophages, cytotoxic cells, and CD45+ leukocytes. (b) Direct comparison between SS and PG shows increased enrichment of dendritic cells and macrophages in SS, while neutrophil abundance is comparable between conditions. In contrast, B cells are strongly enriched in PG and nearly absent in SS. *p* < 0.05, *p < 0.01, ***p < 0.001; CD, cluster of differentiation.

To infer immune cell composition across disease states, we performed cell type deconvolution analysis using predefined gene signatures. Compared to HCs, SS lesions demonstrated marked enrichment of neutrophils, representing the most elevated cell population, followed by macrophages, cytotoxic cells, and CD45+ leukocytes (Figure 2a). Increased scores were also observed for T cells, CD8^+^ T cells and dendritic cells (DCs).

When SS was compared directly with PG, DCs and macrophages emerged as the most enriched populations in SS, whereas neutrophil scores were comparable between the two conditions (Figure 2b). In contrast, PG samples showed strong enrichment of B cells, which were nearly absent in SS, as well as higher representation of T cells and regulatory T cells. These findings indicate distinct immune cellular architectures underlying SS and PG despite their shared neutrophilic phenotype.

### 3.4 Pathway Analysis Identifies Dominant and Disease‐Specific Interferon Signalling in Sweet Syndrome

**FIGURE 3:**
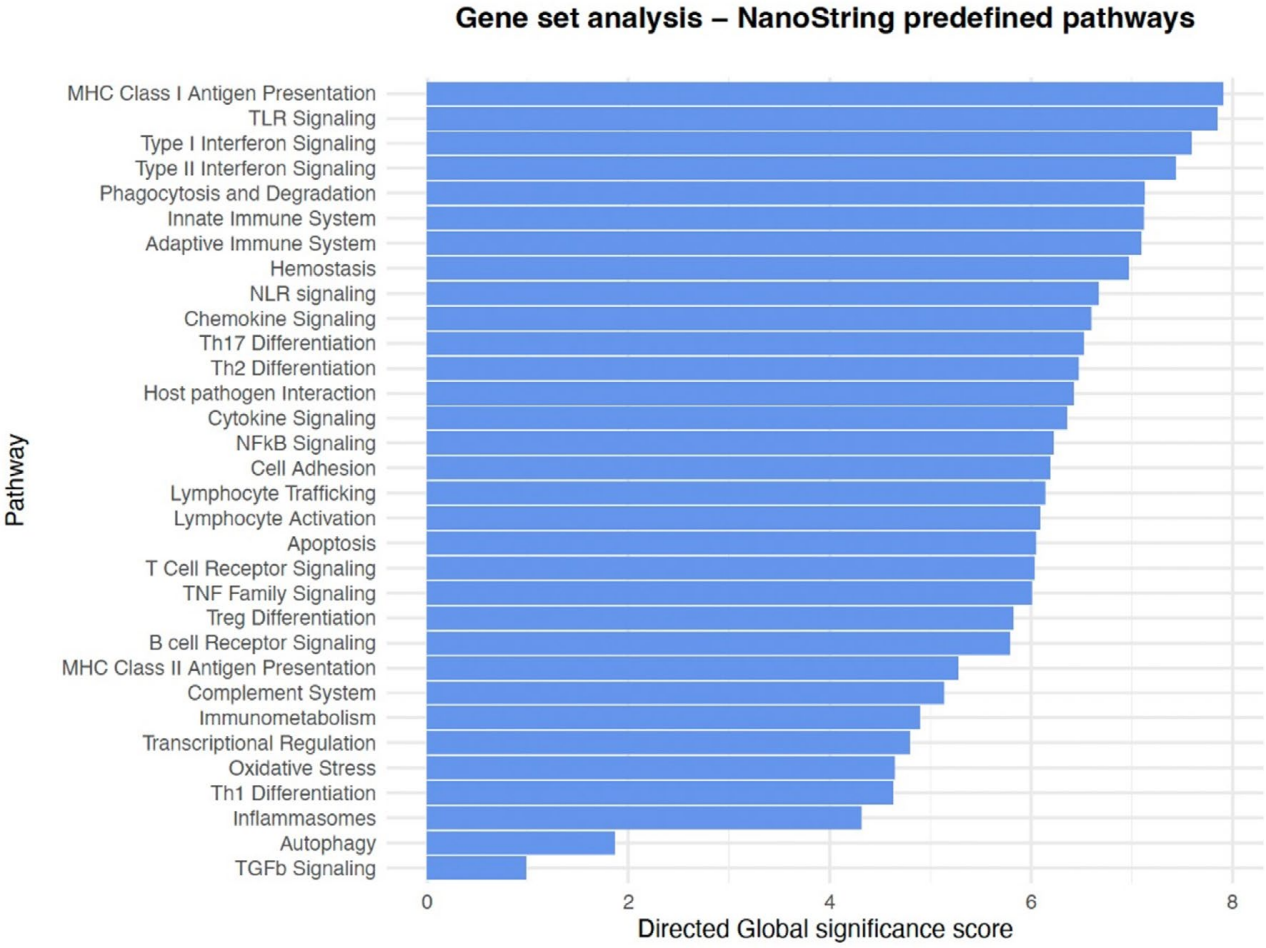
Interferon signalling is a dominant pathway in Sweet syndrome compared with healthy skin. Gene set enrichment analysis comparing SS and HC skin samples. Bar plots depict absolute normalized enrichment scores for significantly altered hallmark pathways. Type I interferon signalling ranks among the most enriched pathways in SS, together with MHC class I antigen presentation, Toll‐like receptor signalling, and type II interferon signalling. IFN, interferon; MHC, major histocompatibility complex; TLR, toll like receptor.

To contextualize differential gene expression at the pathway level, we performed gene set analysis using NanoString‐defined pathways and calculated directed global significance scores. In comparisons between SS and HCs, type I interferon (IFN‐I) signalling emerged among the most strongly enriched pathways, alongside MHC Class I antigen presentation, Toll‐like receptor signalling, and type II interferon signalling (Figure 3), highlighting a dominant interferon‐associated transcriptional programme in SS.

**FIGURE 4:**
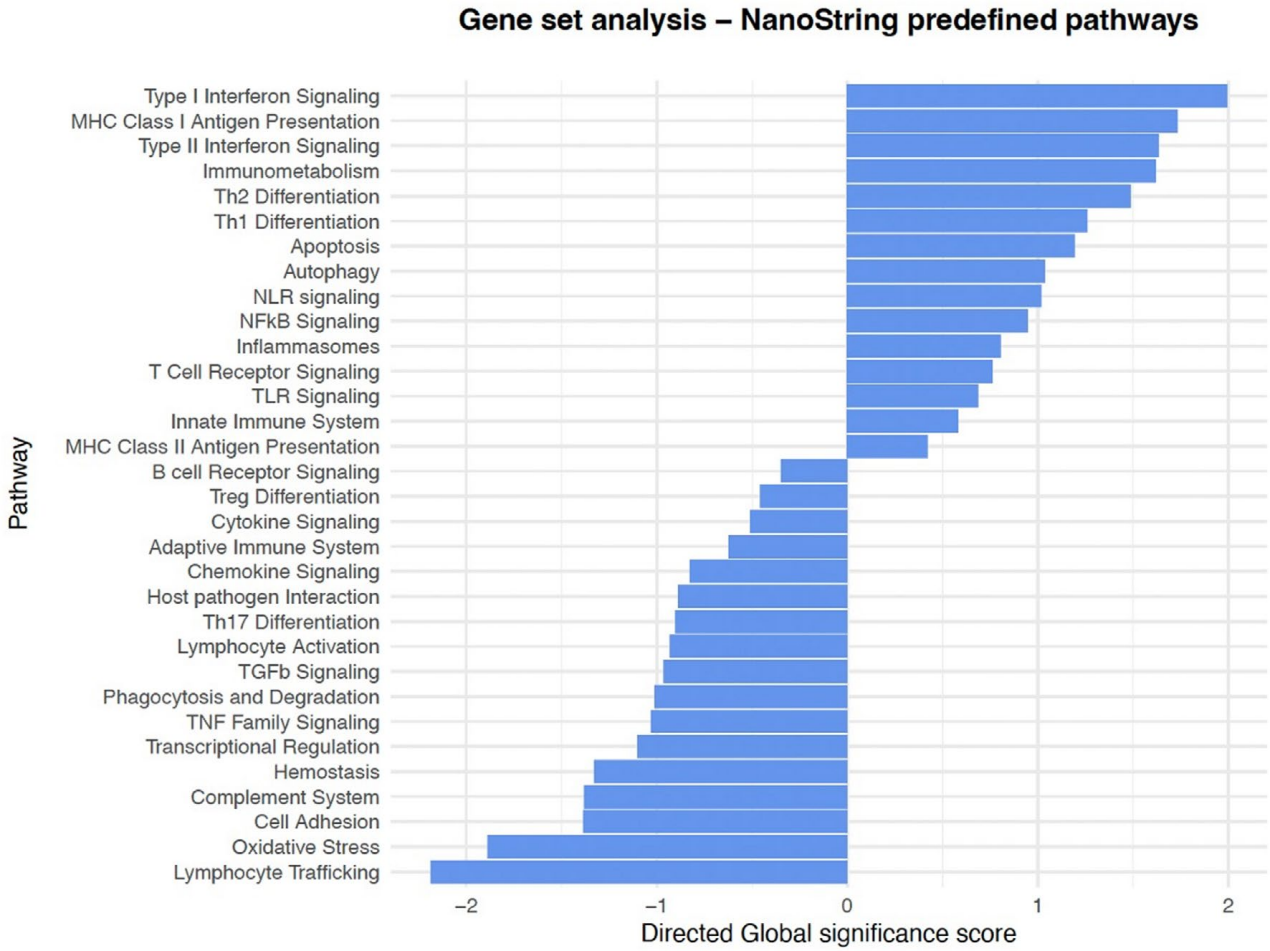
Interferon signalling distinguishes Sweet syndrome from pyoderma gangrenosum. Gene set enrichment analysis comparing SS and PG skin samples. Bar plots show absolute normalized enrichment scores for significantly different hallmark pathways. Type I and II interferon signalling retains a positive directed significance score in SS, indicating higher transcriptional activity relative to PG.

When SS was compared directly with PG, overall pathway enrichment was more attenuated; however, interferon signalling retained a positive directed significance score, indicating sustained transcriptional activation of this pathway in SS relative to PG (Figure 4). These results support IFN‐I signalling as a defining feature of SS within the neutrophilic dermatosis spectrum analysed here.

### 3.5 Unsupervised Clustering Identifies a SS‐Dominant Cluster Characterized by Type I and II Interferon Signalling

**FIGURE 5:**
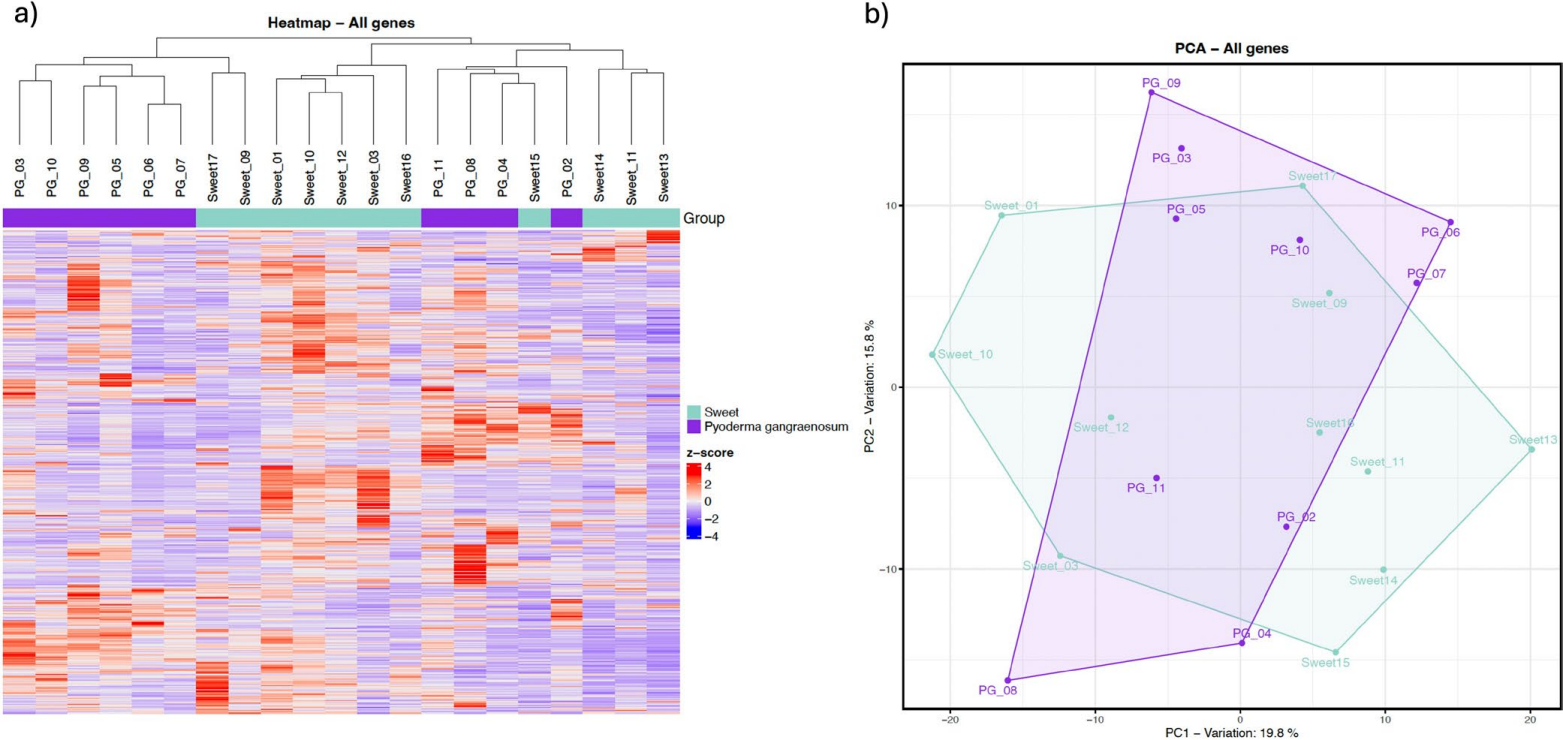
Transcriptional comparison of Sweet syndrome and pyoderma gangrenosum reveals shared and distinct gene expression patterns. (a) Unsupervised hierarchical clustering of gene expression profiles from skin samples of SS and PG shows partial overlap between the two conditions alongside distinct clustering patterns. (b) PCA of SS and PG demonstrates partial convergence with overall separation, supporting both shared inflammatory features and disease‐specific transcriptional programmes.

As shown in Figure 5b, unsupervised hierarchical clustering of SS and PG samples segregated patients into three transcriptionally distinct subgroups (Cluster 1, 2 and 3). Cluster 2 was composed exclusively of SS patients (*n* = 5), whereas Clusters 1 and 3 comprised a mixed population of SS and PG cases. To further investigate the specificity of the observed interferon signature, we compared Cluster 2 (SS‐dominant) with Clusters 1 and 3, using gene set enrichment analysis (GSEA) and cell type deconvolution (Figure S2). Interestingly, GSEA on the NanoString predefined pathways showed that type I and type II Interferon Signalling were the only gene sets reaching FDR‐corrected significance (FDR/*p* < 0.05) in Cluster 2 relative to Clusters 1 + 3. Moreover, cell type deconvolution revealed a consistent enrichment of different cell populations (Th1, CD8+ T cells, etc.), including DCs, in Cluster 2. Although not reaching statistical significance, likely owing to the limited sample size of the SS‐dominant cluster, the magnitude of DC enrichment paralleled that of the significantly increased Th1 cells (*p* < 0.05), CD8^+^ T cells (*p* < 0.05), and cytotoxic cells (*p* < 0.01), while B cells were significantly reduced in SS vs. PG samples (*p* < 0.05). Taken together, these findings indicate that the interferon transcriptional programme is confined to a SS‐dominant subgroup and is not shared by mixed SS/PG clusters. This pathway can therefore be regarded as a disease‐specific immunological signature of SS, even if not uniformly expressed across all patients, a feature that may account for the well‐recognized clinical and biological heterogeneity of the disease.

### 3.6 Sweet Syndrome Is Associated With Altered Expression of Immunoproteasome‐Related Genes

In light of the prominent interferon signature and given that immunoproteasome subunits are well‐established interferon‐stimulated genes [22], we next examined the expression of ubiquitin‐proteasome system components. SS lesions demonstrated selective upregulation of immunoproteasome components compared with both HCs and PG. Specifically, *PSMB8* was upregulated relative to HCs (Log2FC = 1.48 vs. HCs, *p* = 6.66 × 10^−8^; Log2FC = 0.72 vs. PG, *p* = 0.0499), *PSMB9* (Log2FC = 1.16 vs. HCs, *p* = 1.21 × 10^−6^) and *PSMB10* (Log2FC = 1.15 vs. HCs, *p* = 6.66 × 10^−8^; Log2FC = 0.94 vs. PG, *p* = 0.0582) were significantly increased relative to HCs. In parallel, the constitutive proteasome subunit *PSMB7* was significantly downregulated in SS compared to HCs (Log2FC = −0.28; *p* = 0.0046). No significant downregulation of proteasome‐related genes was observed in the SS vs PG comparison.

## 4 Discussion

Sweet syndrome is a prototypical neutrophilic dermatosis within a heterogeneous group of inflammatory skin disorders unified by sterile neutrophilic infiltration [4]. Although SS shares overlapping clinical and histopathological features with other neutrophilic dermatoses such as PG, the molecular mechanisms that define SS and distinguish it from related entities have remained incompletely understood. In this study, transcriptomic analysis of SS skin lesions reveals a distinct inflammatory programme characterized by prominent type I and II interferon signalling, setting SS apart from both healthy skin and PG and providing new insight into its molecular pathogenesis.

Current models of SS pathogenesis emphasize dysregulated innate immunity and autoinflammatory mechanisms centred on IL‐1 family cytokines and neutrophil recruitment [5, 12, 13, 14]. IL‐1–dependent amplification loops, together with TNF‐α and G‐CSF overexpression in selected clinical contexts such as drug‐induced disease, promote neutrophil production, activation, and tissue infiltration [23]. Chemokines including IL‐8 further contribute to neutrophil chemotaxis, consistent with the neutrophil‐rich infiltrates observed histologically [17]. Adaptive immune polarization toward Th1, Th2 and/or Th17 phenotypes has also been proposed to sustain inflammatory signalling, although reported contributions of IL‐17 remain variable [2, 12, 18, 24, 25]. In line with these paradigms, our data confirm robust activation of *IL1B, IL8* and innate immune sensors, including *TLR1* and *TLR2*. Notably, however, these canonical inflammatory pathways coexist with—and are overshadowed by—a dominant interferon–associated transcriptional programme in SS, particularly driven by type I interferon signalling. Indeed, the marked upregulation of interferon‐stimulated genes, including *CXCL9, CXCL10, GBP1, GBP5, IFIT2* and *IRF7*, distinguishes SS from both healthy skin and PG and suggests that sustained interferon signalling represents a defining molecular feature of the disease.

This finding expands existing pathogenetic data and aligns SS with a growing group of inflammatory disorders characterized by aberrant interferon pathway activation. Interestingly, our cell type deconvolution analysis revealed enrichment of DCs in SS lesions– most likely plasmacytoid DCs– offering a plausible cellular source for the sustained production of IFN‐I. Plasmacytoid DCs are specialized producers of type I interferons and have been implicated in autoimmune and autoinflammatory diseases marked by interferon‐driven inflammation. These findings are consistent with recent reports demonstrating increased pDCs and IFN‐I–mediated activation of dermal fibroblasts in SS, supporting a pathogenic circuit linking IFN‐I production to neutrophil recruitment [25, 26].

Notably, beyond the IFN‐I response, our data also revealed activation of the IFN‐II pathway. IFN‐γ, with its broad immunomodulatory functions, may contribute to multiple aspects of SS pathogenesis, including, but not limited to, immunoproteasome induction and enhanced MHC class I antigen presentation [22, 27]. Indeed, in parallel with interferon pathway activation, we observed a selective transcriptional upregulation of the immunoproteasome subunits PSMB8, PSMB9 and PSMB10, accompanied by downregulation of the constitutive subunit PSMB7. These findings might be compatible with an IFN‐driven induction of the immunoproteasome and enhanced MHC class I antigen presentation, as supported by the concurrent enrichment of the MHC class I pathway in our GSEA. As both IFN‐I and IFN‐II are established inducers of immunoproteasome subunits, the two interferon axes may converge on this pathway in SS [28]. Therefore, our findings may align with the emerging concept of ‘MHC‐I‐opathies’, a group of disorders, including Behçet's disease, in which aberrant MHC class I‐driven inflammation appears to play a central role, with the immunoproteasome shaping the antigenic peptide repertoire [29]. Notewhorty, SS has been linked to specific HLA class I alleles, particularly HLA‐B54 and HLA‐Cw1, especially in Japanese cohorts [30], supporting its potential placement within this spectrum. Alongside this, an immunoproteasome‐IFN axis has also been described in autoinflammatory disorders involving altered immunoproteasome pathways, such as CANDLE/PRAAS and VEXAS syndrome [31]. The clinical and histopathological similarities between SS and VEXAS may suggest that altered immunoproteasome activity could contribute, in different ways, to a comparable IFN‐driven neutrophilic inflammatory phenotype, a finding worthy of further investigation.

Importantly, comparison with PG revealed that although both diseases share neutrophilic and inflammatory features, SS is distinguished by a more pronounced interferon‐associated transcriptional programme and a distinct immune cellular architecture. In contrast to PG, which showed enrichment of B cells and regulatory T cell populations, SS lesions were characterized by increased dendritic cell and macrophage signatures, highlighting fundamental differences in immune organization despite sometimes overlapping clinical phenotypes.

From a therapeutic perspective, these findings have direct implications. While systemic corticosteroids remain the mainstay of SS treatment, the identification of a dominant interferon signature suggests that targeted inhibition of the IFN–JAK/STAT axis may represent a rational therapeutic strategy, particularly in refractory disease [32]. The complete resolution achieved by one of our patients included in the analysis, who was treated with the pan‐JAK inhibitor tofacitinib (Table S3), together with documented literature reports of clinical responses to JAK inhibitors in selected SS cases, further supports this possibility [32, 33]. In addition, modulation of proteasome function may represent an alternative or complementary therapeutic avenue in interferon‐driven subsets of SS.

Taken together, our data identify interferon signalling, mostly IFN‐I, as a relevant feature of SS, where interferon activation, dendritic cell enrichment and immunoproteasome remodelling might converge on an IFN‐immunoproteasome‐MHC class I axis. This profile suggests that SS may share pathogenic ground with MHC class I‐driven inflammatory disorders or autoinflammatory conditions linked to altered immunoproteasome pathways. Although further validation will be required, these findings provide a preliminary framework for disease stratification and for the development of targeted therapies in SS.

## Author Contributions

Author order was assigned alphabetically. Data curation: L.C., C.M., M.R., P.‐C.S. and M.N.‐M.; formal analysis: M.N.‐M. and P.‐C.S.; funding acquisition: L.E.F.; investigation: L.C., C.M., M.R., T.K.S., R.A. and Z.F.; methodology: K.K., M.N.‐M., P.‐C.S., R.A. and Z.F.; project administration: L.E.F., T.K.S., L.C., C.M. and M.R.; resources: L.E.F.; software: M.N.‐M. and P.‐C.S.; supervision: L.E.F., T.K.S., P.R. and A.V.M.; validation: L.E.F. and T.K.S.; visualization: L.E.F. and T.K.S.; writing – original draft preparation: L.C., C.M., M.R. and M.D.; writing – review and editing: L.C., C.M., M.R, T.K.S. and L.E.F.

## Funding

This research was partially supported by the Italian Ministry of Health (Ricerca Corrente) of Fondazione IRCCS Ca′ Granda Ospedale Maggiore Policlinico, Milan (Italy) and by the European Union (EU)—Next Generation European Union, Mission 4 Component 2 Inv. 1.5 CUP B63C22000680007. The funders had no role in the study design; in the data collection, analysis and interpretation of data; in the writing of the report and in the decision to submit the article for publication. The researchers were independent from funders.

## Ethics Statement

This study was approved by the Medical Ethics Committee of Ludwig‐Maximilians‐Universität in Munich, Germany (Project No. 22‐0342).

## Conflicts of Interest

The authors declare no conflicts of interest.

## Supporting information


**Table S1:** Differential gene expression analysis of Sweet Syndrome versus healthy controls (HCs). Table listing differentially expressed genes identified by NanoString analysis in skin samples from patients with SS versus HCs, including log_2_ fold change, adjusted *p* values, and false discovery rate–corrected significance. The data demonstrate a robust inflammatory transcriptional signature associated with SS.

**Table S2:** Sweet syndrome–specific transcriptional signatures in comparison with pyoderma gangrenosum and healthy controls. Table summarizing differential gene expression analyses comparing SS with PG and HC skin samples. The table highlights a subset of genes selectively upregulated in SS, including interferon‐related genes that distinguish SS from both PG and healthy controls.

**Table S3:** Clinical and demographics features of patients with Sweet Syndrome.
Table detailing demographic data, clinical features, disease subtype, associated conditions, and treatment information for patients included in the Sweet syndrome cohort.

**Figure S1:** Heatmap and PCA of gene expression profiles from skin samples of SS, PG and healthy controls. (a) Unsupervised clustering based on gene expression profile showed partial overlap, but overall segregation between the SS and PG samples; (b) The PCA plot similarly demonstrated partial convergence with preserved separation, supporting molecular relatedness between SS and PG while highlighting distinct underlying programmes.
**Figure S2:** GSEA from preidentifed Cluster 2 (Sweet‐dominant) vs. Cluster 1 + 3 (combined SS/PG). (a) GSEA revealed that Type I and Type II Interferon Signalling are the only two pathways reaching FDR significance in Cluster 2 relative to the other clusters. (b) Cell type deconvolution revealed a consistent enrichment of different cell populations (Th1, CD8+ T cells, etc.), including DCs, in Cluster 2 vs. Cluster 1 + 3. This figure was generated with the assistance of Claude (claude‐sonnet‐4‐6, Anthropic, 2026).

## Data Availability

Data related to this article can be made available from the corresponding author upon reasonable request.

## References

[1] R. D. Sweet , “An Acute Febrile Neutrophilic Dermatosis,” British Journal of Dermatology 76 (1964): 349–356. 14201182 10.1111/j.1365-2133.1964.tb14541.x

[2] T. P. Joshi , S. K. Friske , D. A. Hsiou , and M. Duvic , “New Practical Aspects of Sweet Syndrome,” American Journal of Clinical Dermatology 23 (2022): 301–318. 35157247 10.1007/s40257-022-00673-4 PMC8853033

[3] L. Calabrese , T. K. Satoh , R. Aoki , et al., “Sweet Syndrome: An Update on Clinical Aspects, Pathophysiology, and Treatment,” Italian Journal of Dermatology and Venereology 159 (2024): 645–662. 39560338 10.23736/S2784-8671.24.07956-8

[4] A. V. Marzano , A. Borghi , D. Wallach , and M. Cugno , “A Comprehensive Review of Neutrophilic Diseases,” Clinical Reviews in Allergy and Immunology 54 (2018): 114–130. 28688013 10.1007/s12016-017-8621-8

[5] E. H. Weiss , C. J. Ko , T. H. Leung , et al., “Neutrophilic Dermatoses: A Clinical Update,” Current Dermatology Reports 11 (2022): 89–102. 35310367 10.1007/s13671-022-00355-8 PMC8924564

[6] C. A. Maronese , F. Derlino , C. Moltrasio , D. Cattaneo , A. Iurlo , and A. V. Marzano , “Neutrophilic and Eosinophilic Dermatoses Associated With Hematological Malignancy,” Frontiers in Medicine (Lausanne) 10 (2023): 1324258. 10.3389/fmed.2023.1324258 PMC10796805 38249974

[7] Q. S. Cook , C. J. Zdanski , C. N. Burkhart , P. B. Googe , P. Thompson , and E. Y. Wu , “Idiopathic, Refractory Sweet's Syndrome Associated With Common Variable Immunodeficiency: A Case Report and Literature Review,” Current Allergy and Asthma Reports 19 (2019): 32. 31089823 10.1007/s11882-019-0864-4

[8] S. Kiratikanon , P. Phinyo , R. Rujiwetpongstorn , et al., “Adult‐Onset Immunodeficiency due to Anti‐Interferon‐Gamma Autoantibody‐Associated Sweet Syndrome: A Distinctive Entity,” Journal of Dermatology 49 (2022): 133–141. 34676591 10.1111/1346-8138.16202

[9] D. F. Thompson and K. E. Montarella , “Drug‐Induced Sweet's Syndrome,” Annals of Pharmacotherapy 41 (2007): 802–811. 17426076 10.1345/aph.1H563

[10] Y. Jia , B. Fu , L. Dong , and M. Zhao , “Sweet Syndrome Induced by SARS‐CoV‐2 Vaccines: A Systematic Review of Patient‐Report Studies,” Human Vaccines & Immunotherapeutics 19 (2023): 2217076. 37313726 10.1080/21645515.2023.2217076 PMC10269388

[11] C. A. Maronese , G. A. Croci , F. Derlino , I. F. Aromolo , C. Moltrasio , and A. V. Marzano , “A Unique Case of Steroid‐Resistant, Giant Cellulitis‐Like Sweet Syndrome Mimicking Alpha‐1‐Antitrypsin Deficiency‐Associated Panniculitis: Successful Treatment With Dapsone,” Clinical and Experimental Dermatology 50 (2025): 1032–1034. 39566187 10.1093/ced/llae499

[12] M. S. Heath and A. G. Ortega‐Loayza , “Insights Into the Pathogenesis of Sweet's Syndrome,” Frontiers in Immunology 10 (2019): 414. 30930894 10.3389/fimmu.2019.00414 PMC6424218

[13] L. Calabrese , F. Ney , R. Aoki , et al., “Characterisation of IL‐1 Family Members in Sweet Syndrome Highlights the Overexpression of IL‐1β and IL‐1R3 as Possible Therapeutic Targets,” Experimental Dermatology 32 (2023): 1915–1923. 37724787 10.1111/exd.14916

[14] L. Calabrese , M. Romagnuolo , M. D'Onghia , P. Rubegni , A. V. Marzano , and C. Moltrasio , “Molecular Characteristics of Sweet Syndrome: A Systematic Review,” Experimental Dermatology 33 (2024): e70022. 39704328 10.1111/exd.70022 PMC11660222

[15] A. V. Marzano , D. Fanoni , E. Antiga , et al., “Expression of Cytokines, Chemokines and Other Effector Molecules in Two Prototypic Autoinflammatory Skin Diseases, Pyoderma Gangrenosum and Sweet's Syndrome,” Clinical and Experimental Immunology 178 (2014): 48–56. 10.1111/cei.12394 PMC4360193 24903614

[16] A. V. Marzano , M. Cugno , V. Trevisan , et al., “Role of Inflammatory Cells, Cytokines and Matrix Metalloproteinases in Neutrophil‐Mediated Skin Diseases,” Clinical and Experimental Immunology 162 (2010): 100–107. 20636397 10.1111/j.1365-2249.2010.04201.x PMC2990935

[17] A. V. Marzano , M. Cugno , V. Trevisan , et al., “Inflammatory Cells, Cytokines and Matrix Metalloproteinases in Amicrobial Pustulosis of the Folds and Other Neutrophilic Dermatoses,” International Journal of Immunopathology and Pharmacology 24 (2011): 451–460. 21658319 10.1177/039463201102400218

[18] E. Antiga , R. Maglie , W. Volpi , et al., “T Helper Type 1‐Related Molecules as Well as Interleukin‐15 Are Hyperexpressed in the Skin Lesions of Patients With Pyoderma Gangrenosum,” Clinical and Experimental Immunology 189 (2017): 383–391. 28518224 10.1111/cei.12989 PMC5543499

[19] P. von den Driesch , “Sweet's Syndrome (Acute Febrile Neutrophilic Dermatosis),” Journal of the American Academy of Dermatology 31 (1994): 535–556. 8089280 10.1016/s0190-9622(94)70215-2

[20] F. Jockenhofer , U. Wollina , K. A. Salva , S. Benson , and J. Dissemond , “The PARACELSUS Score: A Novel Diagnostic Tool for Pyoderma Gangrenosum,” British Journal of Dermatology 180 (2019): 615–620. 29388188 10.1111/bjd.16401

[21] W. Ma , G. Huang , Z. Wang , L. Wang , and Q. Gao , “IRF7: Role and Regulation in Immunity and Autoimmunity,” Frontiers in Immunology 14 (2023): 1236923. 37638030 10.3389/fimmu.2023.1236923 PMC10449649

[22] M. Groettrup , C. J. Kirk , and M. Basler , “Proteasomes in Immune Cells: More Than Peptide Producers?,” Nature Reviews. Immunology 10 (2010): 73–78. 10.1038/nri2687 20010787

[23] V. Durand , Y. Renaudineau , J. O. Pers , P. Youinou , and C. Jamin , “Cross‐Linking of Human FcgammaRIIIb Induces the Production of Granulocyte Colony‐Stimulating Factor and Granulocyte‐Macrophage Colony‐Stimulating Factor by Polymorphonuclear Neutrophils,” Journal of Immunology 167 (2001): 3996–4007. 10.4049/jimmunol.167.7.3996 11564819

[24] R. Stalder , N. Brembilla , C. Conrad , et al., “Interleukin‐17E, Inducible Nitric Oxide Synthase and arginase1 as New Biomarkers in the Identification of Neutrophilic Dermatoses,” Clinical and Experimental Dermatology 47 (2022): 675–683. 34669971 10.1111/ced.14988 PMC9300036

[25] K. J. Cavagnero , J. Albright , F. Li , et al., “Positionally Distinct Interferon‐Stimulated Dermal Immune‐Acting Fibroblasts Promote Neutrophil Recruitment in Sweet Syndrome,” Journal of Allergy and Clinical Immunology 156 (2025): 966–979.e968. 40516577 10.1016/j.jaci.2025.05.029 PMC12678022

[26] M. Swiecki and M. Colonna , “The Multifaceted Biology of Plasmacytoid Dendritic Cells,” Nature Reviews. Immunology 15 (2015): 471–485. 10.1038/nri3865 PMC4808588 26160613

[27] K. Schroder , P. J. Hertzog , T. Ravasi , and D. A. Hume , “Interferon‐Gamma: An Overview of Signals, Mechanisms and Functions,” Journal of Leukocyte Biology 75 (2004): 163–189. 14525967 10.1189/jlb.0603252

[28] Z. Zou , Y. Hao , Z. Tao , et al., “Current Landscape of the Immunoproteasome: Implications for Disease and Therapy,” Cell Death Discov 11 (2025): 406. 40855068 10.1038/s41420-025-02698-0 PMC12378795

[29] M. Giza , D. Koftori , L. Chen , and P. Bowness , “Is Behcet's Disease a ‘Class 1‐Opathy’? The Role of HLA‐B*51 in the Pathogenesis of Behcet's Disease,” Clinical and Experimental Immunology 191 (2018): 11–18. 28898393 10.1111/cei.13049 PMC5721257

[30] H. Takahama and T. Kanbe , “Neutrophilic Dermatosis of the Dorsal Hands: A Case Showing HLA B54, the Marker of Sweet's Syndrome,” International Journal of Dermatology 49 (2010): 1079–1080. 20883277 10.1111/j.1365-4632.2009.04422.x

[31] D. B. Beck , A. Werner , D. L. Kastner , and I. Aksentijevich , “Disorders of Ubiquitylation: Unchained Inflammation,” Nature Reviews Rheumatology 18 (2022): 435–447. 35523963 10.1038/s41584-022-00778-4 PMC9075716

[32] Y. Nousari , B. C. Wu , and G. Valenzuela , “Successful Use of Baricitinib in the Treatment of Refractory Rheumatoid Arthritis‐Associated Sweet Syndrome,” Clinical and Experimental Dermatology 46 (2021): 1330–1332. 33914946 10.1111/ced.14712

[33] V. Duque‐Clavijo , H. Q. Doan , J. A. Das , and S. K. Tyring , “Sweet Syndrome Successfully Treated With Upadacitinib,” JAAD Case Reports 65 (2025): 179–182. 41143222 10.1016/j.jdcr.2025.08.003 PMC12547819

