# Supplementary material for: Type I and II Interferon Signalling Characterizes the Transcriptional Landscape of Sweet Syndrome
Source: Exp Dermatol. 2026 Jul 6;35(7):e70323. doi: 10.1111/exd.70323 (PMC13338580; doi:10.1111/exd.70323)
Supplement: Supplementary file 1 — Table S1: Differential gene expression analysis of Sweet Syndrome versus healthy controls (HCs). Table listing differentially expressed genes identified by NanoString analysis in skin samples from patients with SS versus HCs, including log2 fold change, adjusted p values, and false discovery rate–corrected significance. The data demonstrate a robust inflammatory transcriptional signature associated with SS. [file EXD-35-e70323-s003.pdf]

Suppl. Table 1. Differential gene expression analysis of Sweet Syndrome versus healthy controls.								
	Log2FC	StdError	stat	LowerConfLimit	UpperConfLimit	pvalue	pvalue.adj	method
CXCL10	7,6288151	0,76952	9,91377	6,120561315	9,137068851	3,82E-10	2,92E-09	lm.nb
CXCL9	6,9790855	0,68137	10,2428	5,643607598	8,314563352	1,97E-10	1,65E-09	lm.nb
GBP5	6,5928396	0,63623	10,3624	5,345833488	7,839845698	1,56E-10	1,36E-09	lm.nb
IL8	6,3917985	1,02666	6,22579	4,379536662	8,404060373	1,64E-06	4,04E-06	loglinear
LILRA5	5,792208	0,47849	12,1051	4,854357873	6,730058193	5,96E-12	1,12E-10	lm.nb
IL1B	5,5239281	0,73209	7,54544	4,089034731	6,9588215	6,71E-08	2,26E-07	lm.nb
S100A9	5,4057392	0,43404	12,4545	4,55502112	6,256457271	3,22E-12	6,34E-11	lm.nb
ITGAX	5,078828	0,32244	15,7514	4,446851375	5,710804699	1,73E-14	1,33E-12	lm.nb
CCRL2	5,0605016	0,45649	11,0858	4,165787784	5,955215448	3,85E-11	4,83E-10	lm.nb
S100A8	4,9715619	0,50066	9,93003	3,990270175	5,952853663	3,69E-10	2,88E-09	lm.nb
GBP1	4,7723	0,44689	10,6789	3,896393403	5,648206666	8,38E-11	9,18E-10	lm.nb
LILRB3	4,748061	0,34735	13,6695	4,067261407	5,428860654	4,19E-13	1,26E-11	lm.nb
IFIT2	4,7095378	0,6224	7,5667	3,489627423	5,929448109	6,39E-08	2,19E-07	lm.nb
CCL8	4,5693656	0,7006	6,5221	3,196194447	5,942536844	7,85E-07	2,09E-06	lm.nb
GZMB	4,5675354	0,35614	12,8252	3,869503862	5,265566996	1,70E-12	3,85E-11	lm.nb
CLEC4E	4,4553744	0,54328	8,20091	3,390549235	5,520199525	1,49E-08	6,30E-08	lm.nb
LILRA2	4,4004935	0,30601	14,3802	3,800713453	5,000273592	1,36E-13	5,10E-12	lm.nb
CXCL1	4,3597929	0,3775	11,549	3,619883397	5,099702392	1,63E-11	2,45E-10	lm.nb
GNLY	4,1154303	0,53297	7,72175	3,070817212	5,160043372	4,45E-08	1,62E-07	lm.nb
LILRB2	4,1038691	0,22061	18,6023	3,671470858	4,536267245	3,71E-16	5,59E-14	lm.nb
SLAMF7	4,1009529	0,45526	9,00801	3,208650777	4,993254942	2,53E-09	1,39E-08	lm.nb
CLEC5A	4,002254	0,53363	7,50005	2,956338243	5,048169677	7,46E-08	2,48E-07	lm.nb
IRF7	3,9508524	0,37348	10,5786	3,218838337	4,682866364	1,02E-10	9,79E-10	lm.nb
TLR2	3,8715092	0,23319	16,6022	3,414450972	4,328567337	5,19E-15	5,86E-13	lm.nb
CCL4	3,8596649	0,53274	7,24494	2,815495544	4,903834227	1,36E-07	4,14E-07	lm.nb
MX1	3,8239459	0,4461	8,57193	2,949588503	4,698303366	6,52E-09	3,07E-08	lm.nb
CSF3R	3,7259584	0,34976	10,653	3,0404336	4,411483136	8,81E-11	9,18E-10	lm.nb
CCL19	3,6615892	0,39732	9,21565	2,882836352	4,440342115	1,62E-09	9,53E-09	lm.nb
SELL	3,59623	0,36599	9,8261	2,878894715	4,313565373	4,56E-10	3,33E-09	lm.nb
CCR5	3,5838216	0,40019	8,95527	2,79944634	4,368196818	2,83E-09	1,51E-08	lm.nb
PLAUR	3,5197393	0,36765	9,57371	2,799152389	4,240326213	7,67E-10	5,11E-09	lm.nb
LILRA6	3,5119831	0,27659	12,6973	2,969861856	4,054104255	2,12E-12	4,36E-11	lm.nb
IRF1	3,4352203	0,35888	9,57195	2,731807645	4,13863287	7,70E-10	5,11E-09	lm.nb
FCGR2A	3,4268147	0,24116	14,2098	2,954142818	3,899486516	1,77E-13	6,15E-12	lm.nb
CLEC7A	3,3888852	0,21533	15,7384	2,966845331	3,810925092	1,76E-14	1,33E-12	lm.nb
TNFSF15	3,2962154	0,48996	6,72754	2,335896328	4,256534465	4,74E-07	1,31E-06	lm.nb
CCR1	3,2867244	0,21617	15,2043	2,863029494	3,710419273	3,86E-14	2,18E-12	lm.nb
FCER1G	3,2688872	0,24557	13,3114	2,787569227	3,750205234	7,54E-13	2,00E-11	lm.nb
ITGA4	3,2534311	0,32881	9,89466	2,608969599	3,897892623	3,97E-10	2,94E-09	lm.nb
LILRB4	3,2079092	0,34576	9,27775	2,530212439	3,885606033	1,42E-09	8,58E-09	lm.nb
PTAFR	3,175812	0,21829	14,5489	2,747971337	3,60365265	1,04E-13	5,10E-12	lm.nb
CR1	3,1751154	0,33775	9,40071	2,513120336	3,837110462	1,10E-09	6,81E-09	lm.nb

CLEC4A	3,1615621	0,28178	11,2201	2,609281133	3,713843163	2,99E-11	3,98E-10	lm.nb
FCGR3AB	3,1401564	0,41153	7,6304	2,333553298	3,946759594	5,50E-08	1,93E-07	lm.nb
SELPLG	3,0941667	0,29177	10,6047	2,522290542	3,66604288	9,68E-11	9,72E-10	lm.nb
JAK3	3,084996	0,20058	15,3807	2,691866865	3,478125051	2,97E-14	1,92E-12	lm.nb
DEFB103B	3,0791065	0,7029	4,38058	1,701424492	4,456788598	0,00019	0,000326	lm.nb
CSF2RB	3,0690328	0,22446	13,6727	2,629081908	3,508983621	4,17E-13	1,26E-11	lm.nb
LCP2	3,0158407	0,23703	12,7234	2,55126026	3,48042111	2,03E-12	4,36E-11	lm.nb
CCL2	3,0019081	0,44844	6,69405	2,122956902	3,88085936	5,15E-07	1,41E-06	lm.nb
CIITA	2,936817	0,30621	9,59076	2,336639147	3,53699491	7,40E-10	5,11E-09	lm.nb
S100A7	2,8995639	0,5398	5,37155	1,841554696	3,9575731	1,43E-05	2,88E-05	lm.nb
TLR8	2,8882705	0,27293	10,5824	2,353327187	3,423213876	1,01E-10	9,79E-10	lm.nb
OAS1	2,8722545	0,37636	7,63174	2,134596217	3,609912872	5,49E-08	1,93E-07	lm.nb
BCL3	2,8700084	0,26958	10,6464	2,341638927	3,398377785	8,93E-11	9,18E-10	lm.nb
CD45RB	2,8690558	0,22125	12,9674	2,435404605	3,302707093	1,34E-12	3,19E-11	lm.nb
PSMB10	2,8582815	0,2469	11,5766	2,374355056	3,342207924	1,55E-11	2,41E-10	lm.nb
CD53	2,8170379	0,1958	14,387	2,433261987	3,200813776	1,34E-13	5,10E-12	lm.nb
CYBB	2,796476	0,26929	10,3847	2,268673013	3,324279	1,49E-10	1,35E-09	lm.nb
STAT1	2,7775586	0,29111	9,54132	2,206986001	3,348131132	8,20E-10	5,29E-09	lm.nb
PLAU	2,7745488	0,28031	9,8982	2,225144019	3,323953535	3,94E-10	2,94E-09	lm.nb
CD86	2,7647364	0,37026	7,46706	2,039031035	3,49044182	8,07E-08	2,60E-07	lm.nb
IL1RN	2,7484368	0,34007	8,08193	2,081896175	3,414977379	1,95E-08	8,01E-08	lm.nb
ITGB2	2,747434	0,23414	11,7343	2,288526437	3,206341657	1,16E-11	1,94E-10	lm.nb
BATF3	2,7433994	0,33593	8,1666	2,084978413	3,401820338	1,61E-08	6,74E-08	lm.nb
ICAM1	2,6517408	0,33904	7,82139	1,98722809	3,316253501	3,54E-08	1,34E-07	lm.nb
CD247	2,639031	0,35557	7,42197	1,942113502	3,335948538	8,97E-08	2,85E-07	lm.nb
MLKL	2,5594906	0,23607	10,8421	2,096794636	3,02218649	6,12E-11	7,09E-10	lm.nb
TNFSF13B	2,5231793	0,27545	9,16034	1,983305029	3,06305361	1,83E-09	1,06E-08	lm.nb
FCGR2AC	2,5030002	0,23239	10,7708	2,047519534	2,958480789	7,02E-11	7,93E-10	lm.nb
CCR2	2,4998499	0,27517	9,0847	1,960513804	3,039186036	2,14E-09	1,21E-08	lm.nb
PTPRC_all	2,4970196	0,1735	14,3921	2,156959805	2,83707949	1,33E-13	5,10E-12	lm.nb
IFI35	2,4904773	0,31444	7,92042	1,874179732	3,106774935	2,82E-08	1,12E-07	lm.nb
HAVCR2	2,4900786	0,28139	8,84924	1,938556064	3,041601158	3,56E-09	1,83E-08	lm.nb
IRF5	2,4665678	0,26722	9,23047	1,942816259	2,99031934	1,57E-09	9,36E-09	lm.nb
IL2RA	2,4478717	0,47186	5,18776	1,523035754	3,372707658	2,29E-05	4,49E-05	lm.nb
CFB	2,442272	0,25948	9,41232	1,933698919	2,950845011	1,07E-09	6,74E-09	lm.nb
PRF1	2,4415453	0,35949	6,79178	1,736954371	3,146136232	4,06E-07	1,13E-06	lm.nb
TLR1	2,4398707	0,11292	21,6078	2,218555341	2,661186029	1,08E-17	4,90E-15	lm.nb
CD163	2,4395406	0,22425	10,8788	2,000014769	2,879066348	5,71E-11	6,79E-10	lm.nb
CCL24	2,4337139	0,59654	4,0797	1,264491663	3,602936108	0,0004	0,000683	lm.nb
TAP2	2,4315376	0,23911	10,1691	1,96288234	2,900192867	2,28E-10	1,81E-09	lm.nb
CD48	2,3898873	0,28042	8,5225	1,840261987	2,939512583	7,27E-09	3,37E-08	lm.nb
LTF	2,3890569	0,58275	4,09966	1,246876258	3,531237582	0,00038	0,000654	lm.nb
ITGAL	2,3776205	0,27561	8,62667	1,837419615	2,917821292	5,78E-09	2,78E-08	lm.nb
LAIR1	2,3641375	0,22713	10,4085	1,918953835	2,809321147	1,42E-10	1,31E-09	lm.nb

TNFAIP6	2,3556263	0,41225	5,71404	1,547611887	3,163640801	5,95E-06	1,33E-05	lm.nb
TNFRSF10C	2,3376778	0,37035	6,31207	1,611790561	3,063564951	1,32E-06	3,32E-06	lm.nb
CD68	2,3375487	0,25547	9,14982	1,83681803	2,838279322	1,87E-09	1,07E-08	lm.nb
IFIH1	2,3357901	0,31431	7,43155	1,719747671	2,951832608	8,77E-08	2,81E-07	lm.nb
TAP1	2,3336341	0,26067	8,95235	1,822715323	2,844552828	2,85E-09	1,51E-08	lm.nb
IRAK2	2,3288944	0,37732	6,1722	1,589347354	3,068441441	1,87E-06	4,53E-06	lm.nb
CTSS	2,3211229	0,25566	9,079	1,820032608	2,822213258	2,17E-09	1,21E-08	lm.nb
PML	2,3016719	0,25785	8,92626	1,796278178	2,80706557	3,01E-09	1,58E-08	lm.nb
ZAP70	2,2953307	0,41717	5,50211	1,477671602	3,112989882	1,02E-05	2,18E-05	lm.nb
PLA2G2A	2,2830618	0,48757	4,68253	1,32742347	3,238700169	8,48E-05	0,000153	lm.nb
SELE	2,2600841	0,47248	4,78347	1,334027403	3,186140814	6,53E-05	0,00012	lm.nb
CARD9	2,2557296	0,28221	7,99318	1,702604234	2,808854976	2,39E-08	9,63E-08	lm.nb
CCL5	2,2521817	0,38499	5,8499	1,497591897	3,006771506	4,22E-06	9,58E-06	lm.nb
IL2RG	2,249385	0,21709	10,3613	1,823880856	2,674889113	1,56E-10	1,36E-09	lm.nb
TNFRSF1B	2,2400625	0,18594	12,0473	1,875622271	2,604502737	6,60E-12	1,19E-10	lm.nb
CD45R0	2,2217023	0,18551	11,9761	1,858098834	2,585305667	7,50E-12	1,30E-10	lm.nb
IL1R2	2,2210989	0,43115	5,15154	1,376039816	3,066157929	2,52E-05	4,91E-05	lm.nb
MSR1	2,2100528	0,30072	7,34926	1,620645821	2,79945976	1,06E-07	3,30E-07	lm.nb
GZMA	2,1857996	0,39224	5,57259	1,41700634	2,954592929	8,54E-06	1,83E-05	lm.nb
IL10	2,1807076	0,40292	5,41226	1,390984101	2,970431077	1,29E-05	2,62E-05	lm.nb
CXCR2	2,1276599	0,28182	7,54977	1,575297157	2,680022589	6,64E-08	2,26E-07	lm.nb
C1QB	2,098531	0,41473	5,06003	1,285666797	2,911395185	3,19E-05	6,11E-05	lm.nb
CD83	2,0981437	0,36792	5,70272	1,377020741	2,819266572	6,13E-06	1,36E-05	lm.nb
CISH	2,0883259	0,26938	7,7522	1,560331676	2,616320104	4,15E-08	1,52E-07	lm.nb
PSMB9	2,0864613	0,2674	7,80277	1,562356845	2,61056579	3,69E-08	1,38E-07	lm.nb
RARRES3	2,0808496	0,37967	5,48071	1,336699912	2,824999235	1,08E-05	2,26E-05	lm.nb
IL7R	2,0645703	0,37783	5,46422	1,324014899	2,805125767	1,13E-05	2,33E-05	lm.nb
CXCR4	2,0460483	0,36484	5,60806	1,330960925	2,761135698	7,80E-06	1,68E-05	lm.nb
TNF	2,04532	0,26248	7,7924	1,530866748	2,559773256	3,78E-08	1,40E-07	lm.nb
LCK	2,0344335	0,43405	4,68706	1,183689344	2,88517765	8,38E-05	0,000152	lm.nb
ITGAM	2,02925	0,19404	10,4581	1,648939166	2,409560877	1,29E-10	1,21E-09	lm.nb
HLA_DRB3	2,0181044	0,27355	7,37751	1,48194996	2,554258823	9,96E-08	3,10E-07	lm.nb
CSF1R	2,0173526	0,27877	7,2366	1,470962043	2,563743144	1,39E-07	4,19E-07	lm.nb
BATF	1,9997167	0,32334	6,18455	1,365969153	2,633464251	1,82E-06	4,41E-06	lm.nb
TAGAP	1,9728484	0,30337	6,50305	1,378238136	2,567458652	8,23E-07	2,16E-06	lm.nb
MYD88	1,942523	0,14559	13,3421	1,657158953	2,227887097	7,16E-13	2,00E-11	lm.nb
CTLA4_all	1,9351809	0,40509	4,77718	1,141207437	2,729154424	6,64E-05	0,000122	lm.nb
NFKB2	1,9227068	0,14491	13,2682	1,638681189	2,206732326	8,10E-13	2,03E-11	lm.nb
TGFB1	1,8893454	0,1659	11,3883	1,564176798	2,214514072	2,19E-11	3,09E-10	lm.nb
TRAF1	1,8717413	0,35489	5,2742	1,176164422	2,567318099	1,84E-05	3,66E-05	lm.nb
CD8A	1,8583567	0,44081	4,21575	0,994363029	2,722350462	0,00028	0,00049	lm.nb
CD14	1,8582708	0,21847	8,50581	1,430068148	2,286473419	7,55E-09	3,45E-08	lm.nb
RUNX1	1,8492346	0,24714	7,4824	1,364831083	2,333638071	7,78E-08	2,54E-07	lm.nb
PTPN6	1,8206763	0,09458	19,2492	1,635290716	2,006061885	1,66E-16	3,76E-14	lm.nb

BTK	1,820483	0,24904	7,3099	1,332357639	2,308608311	1,17E-07	3,59E-07	lm.nb
CD74	1,8189085	0,26552	6,85036	1,298489351	2,339327654	3,52E-07	1,01E-06	lm.nb
IKZF1	1,7979067	0,2627	6,8439	1,283010963	2,3128024	3,57E-07	1,02E-06	lm.nb
CD2	1,7931104	0,46164	3,88421	0,888293335	2,697927492	0,00067	0,001104	lm.nb
IL2RB	1,7870301	0,26291	6,79714	1,271728572	2,302331578	4,00E-07	1,12E-06	lm.nb
SOCS1	1,7816039	0,47353	3,76241	0,853489506	2,709718367	0,00091	0,001463	lm.nb
LTB4R	1,7814315	0,1546	11,5225	1,478407299	2,084455695	1,71E-11	2,49E-10	lm.nb
STAT2	1,7811582	0,21579	8,25432	1,358219473	2,204096861	1,32E-08	5,74E-08	lm.nb
CCL22	1,7792831	0,51151	3,47848	0,776721195	2,781845021	0,00186	0,002836	lm.nb
LAMP3	1,7765087	0,32321	5,49643	1,143014421	2,410003013	1,04E-05	2,20E-05	lm.nb
IFNAR2	1,7612109	0,17264	10,2016	1,422834719	2,09958711	2,14E-10	1,73E-09	lm.nb
IL10RA	1,7508677	0,2189	7,99861	1,321830494	2,179904968	2,36E-08	9,60E-08	lm.nb
LILRA3	1,7293355	0,19847	8,71348	1,340341014	2,118330065	4,78E-09	2,35E-08	lm.nb
FASL	1,725567	0,37514	4,59984	0,990300328	2,460833763	0,00011	0,000189	lm.nb
NCF4	1,6895546	0,16532	10,2199	1,365526114	2,013582994	2,06E-10	1,70E-09	lm.nb
MME	1,6734444	0,40739	4,10775	0,874965011	2,471923884	0,00038	0,000643	lm.nb
ARHGDIB	1,6718759	0,14324	11,6715	1,391116456	1,952635421	1,30E-11	2,10E-10	lm.nb
HLA_DMA	1,6664026	0,25576	6,51555	1,165117095	2,167688073	7,98E-07	2,11E-06	lm.nb
TNFSF10	1,6618325	0,25693	6,46805	1,158251072	2,165413904	8,97E-07	2,33E-06	lm.nb
CFP	1,6162957	0,25189	6,41662	1,122587087	2,110004328	1,02E-06	2,62E-06	lm.nb
RELB	1,604944	0,1889	8,49639	1,2347058	1,975182281	7,71E-09	3,48E-08	lm.nb
CD40	1,6009729	0,21105	7,58587	1,187321545	2,014624226	6,11E-08	2,11E-07	lm.nb
CD5	1,5939646	0,45364	3,51375	0,704837323	2,483091954	0,00171	0,002605	lm.nb
THY1	1,5605743	0,30711	5,08146	0,958636434	2,162512157	3,02E-05	5,83E-05	lm.nb
SOCS3	1,5453928	0,54752	2,82252	0,472250838	2,618534739	0,00921	0,012652	lm.nb
CD27	1,5433616	0,40345	3,82537	0,752592064	2,334131128	0,00077	0,00126	lm.nb
TRAF3	1,541308	0,13555	11,3711	1,275638005	1,806978065	2,26E-11	3,09E-10	lm.nb
ICAM3	1,5270746	0,18745	8,1465	1,15966967	1,894479616	1,69E-08	6,99E-08	lm.nb
CD97	1,5251588	0,179	8,52035	1,174314949	1,876002571	7,31E-09	3,37E-08	lm.nb
IL15	1,5227937	0,26645	5,71509	1,000549193	2,045038166	5,94E-06	1,33E-05	lm.nb
CD3EAP	1,5125104	0,2764	5,47209	0,970757258	2,054263576	1,10E-05	2,30E-05	lm.nb
HLA_DMB	1,4835242	0,26999	5,49468	0,95433831	2,01271003	1,04E-05	2,20E-05	lm.nb
C1QA	1,4810309	0,40164	3,68743	0,693811022	2,268250826	0,0011	0,001733	lm.nb
IL4R	1,4782813	0,18025	8,20144	1,124998209	1,831564341	1,49E-08	6,30E-08	lm.nb
TIGIT	1,4743347	0,45298	3,25476	0,586498063	2,362171303	0,00325	0,004751	lm.nb
IL6R	1,4704528	0,14334	10,2584	1,189503696	1,751401872	1,91E-10	1,63E-09	lm.nb
PTPN22	1,4675799	0,24071	6,09678	0,995780219	1,939379506	2,26E-06	5,35E-06	lm.nb
GZMK	1,4575478	0,44245	3,29426	0,590345387	2,324750202	0,00295	0,004353	lm.nb
LY96	1,4444005	0,2175	6,64106	1,018109251	1,870691698	5,86E-07	1,60E-06	lm.nb
TAPBP	1,4443815	0,15131	9,54557	1,14780524	1,740957682	8,13E-10	5,29E-09	lm.nb
PTGS2	1,4442557	0,57215	2,52427	0,322847303	2,565664114	0,01832	0,024065	lm.nb
IFI16	1,4332204	0,14796	9,68674	1,143224734	1,723215989	6,07E-10	4,36E-09	lm.nb
MIF	1,430739	0,15155	9,44046	1,133693329	1,727784678	1,01E-09	6,44E-09	lm.nb
CD28	1,429108	0,24715	5,78226	0,944686409	1,913529493	5,01E-06	1,13E-05	lm.nb

IFITM1	1,4170706	0,24123	5,87446	0,94426845	1,889872651	3,96E-06	9,09E-06	lm.nb
HLA_DRB1	1,3875301	0,60165	2,3062	0,208290092	2,566770148	0,02967	0,037568	lm.nb
PSMB8	1,3756294	0,17953	7,66256	1,02375847	1,727500352	5,11E-08	1,83E-07	lm.nb
CD244	1,3719128	0,26785	5,12199	0,846931486	1,896894113	2,72E-05	5,27E-05	lm.nb
IL1RL1	1,3697785	0,37492	3,65349	0,634928067	2,104628917	0,0012	0,001863	lm.nb
TMEM173	1,364656	0,15609	8,74252	1,058711408	1,670600668	4,49E-09	2,25E-08	lm.nb
HLA_B	1,3171526	0,19315	6,81918	0,938570766	1,695734455	3,79E-07	1,07E-06	lm.nb
HLA_A	1,2979832	0,20229	6,41659	0,901503796	1,69446266	1,02E-06	2,62E-06	lm.nb
KCNJ2	1,2855445	0,19505	6,59091	0,903250302	1,667838734	6,63E-07	1,78E-06	lm.nb
IRAK3	1,283583	0,23853	5,38126	0,816067249	1,751098792	1,39E-05	2,83E-05	lm.nb
CD3E	1,2828348	0,28751	4,46196	0,719324819	1,8463447	0,00015	0,000266	lm.nb
IKBKE	1,2787561	0,20548	6,22337	0,87602223	1,681489931	1,65E-06	4,05E-06	lm.nb
JAK2	1,2711737	0,20453	6,21501	0,870289582	1,672057871	1,68E-06	4,11E-06	lm.nb
CASP8	1,2434333	0,11127	11,175	1,025344976	1,461521585	3,25E-11	4,20E-10	lm.nb
MAPK11	1,236158	0,21855	5,65621	0,807802453	1,664513643	6,90E-06	1,51E-05	lm.nb
IL1R1	1,2299857	0,13257	9,27795	0,970146881	1,489824451	1,42E-09	8,58E-09	lm.nb
VCAM1	1,2286508	0,28453	4,31819	0,670973869	1,786327664	0,00022	0,000381	lm.nb
TLR4	1,2103088	0,22145	5,46535	0,776264307	1,6443533	1,12E-05	2,33E-05	lm.nb
C4AB	1,2068035	0,32308	3,73531	0,57356761	1,840039384	0,00097	0,001562	lm.nb
TNFSF11	1,2010819	0,35257	3,40662	0,510039067	1,892124763	0,00223	0,003359	lm.nb
IL18R1	1,197921	0,23703	5,05396	0,733349544	1,662492458	3,24E-05	6,18E-05	lm.nb
NLRP3	1,197709	0,4316	2,77503	0,35176997	2,043648074	0,01029	0,0141	lm.nb
C2	1,1934387	0,30884	3,86429	0,588116272	1,798761056	0,0007	0,001157	lm.nb
LTB4R2	1,179362	0,15082	7,81945	0,88374659	1,474977356	3,55E-08	1,34E-07	lm.nb
CCND3	1,1729789	0,12182	9,62876	0,93421091	1,411746909	6,84E-10	4,83E-09	lm.nb
IRF8	1,1723664	0,26415	4,43823	0,65462942	1,690103448	0,00016	0,000282	lm.nb
PDCD1LG2	1,1716201	0,23333	5,02122	0,714286354	1,628953871	3,53E-05	6,64E-05	lm.nb
LITAF	1,1700329	0,15553	7,52291	0,865195449	1,474870276	7,08E-08	2,37E-07	lm.nb
NOD2	1,1546772	0,1707	6,76434	0,82010374	1,489250715	4,34E-07	1,20E-06	lm.nb
HLA_C	1,1369818	0,21573	5,27043	0,714153815	1,559809792	1,85E-05	3,68E-05	lm.nb
SYK	1,111287	0,14983	7,41704	0,817622357	1,404951545	9,07E-08	2,87E-07	lm.nb
HLA_DRA	1,1061379	0,26107	4,23696	0,594442877	1,617832949	0,00027	0,000466	lm.nb
TNFRSF8	1,1014854	0,27934	3,9432	0,553982799	1,648988017	0,00057	0,000956	lm.nb
TNFRSF14	1,097334	0,16699	6,57134	0,770037668	1,424630313	6,95E-07	1,86E-06	lm.nb
PTPN2	1,0956334	0,14294	7,66514	0,815476536	1,37579034	5,08E-08	1,83E-07	lm.nb
BST2	1,0935377	0,35095	3,11596	0,405681238	1,78139426	0,00456	0,006505	lm.nb
TCF7	1,0875091	0,29351	3,70518	0,512228628	1,662789669	0,00105	0,001668	lm.nb
PRKCD	1,0870135	0,12421	8,75148	0,843563682	1,330463281	4,40E-09	2,24E-08	lm.nb
TICAM1	1,0793845	0,19827	5,44389	0,690766202	1,468002843	1,19E-05	2,44E-05	lm.nb
MAP4K1	1,0792714	0,25342	4,25876	0,582560197	1,575982516	0,00025	0,000442	lm.nb
NOD1	1,0735985	0,15242	7,04354	0,774849103	1,372347814	2,21E-07	6,48E-07	lm.nb
CD44	1,0453135	0,13352	7,82883	0,783612263	1,307014709	3,48E-08	1,33E-07	lm.nb
FADD	1,0424873	0,16285	6,40158	0,723304316	1,361670367	1,06E-06	2,70E-06	lm.nb
STAT3	1,0389915	0,1468	7,07748	0,751258789	1,326724157	2,03E-07	6,01E-07	lm.nb

CD209	1,0316667	0,26558	3,88458	0,511129778	1,552203575	0,00067	0,001104	lm.nb
CMKLR1	1,0153808	0,27591	3,68007	0,474589992	1,55617152	0,00112	0,00176	lm.nb
SRC	1,0128198	0,16249	6,23305	0,694335558	1,331304082	1,61E-06	3,99E-06	lm.nb
RIPK3	0,9978865	0,13028	7,6594	0,74253263	1,25324045	5,15E-08	1,83E-07	lm.nb
CD96	0,9913834	0,31103	3,18744	0,381767417	1,600999463	0,00383	0,005534	lm.nb
CD40LG	0,9636702	0,38009	2,53537	0,218693282	1,708647029	0,01786	0,023539	lm.nb
TRAF2	0,9495233	0,10896	8,71474	0,735969603	1,163076954	4,77E-09	2,35E-08	lm.nb
CTSC	0,9395521	0,11209	8,38212	0,719855519	1,159248661	9,93E-09	4,40E-08	lm.nb
HLA_DPA1	0,9154306	0,31042	2,94901	0,307008442	1,52385273	0,00682	0,009548	lm.nb
CD3D	0,9041872	0,3098	2,91866	0,296987888	1,511386432	0,00733	0,010201	lm.nb
MASP1	0,9020248	0,21803	4,13711	0,474680858	1,329368823	0,00035	0,000598	lm.nb
BCL2L11	0,8889786	0,22247	3,99589	0,452930783	1,325026426	0,0005	0,000844	lm.nb
ATG12	0,8880907	0,26083	3,4049	0,376869941	1,399311374	0,00224	0,003363	lm.nb
MAPKAPK2	0,8857039	0,11304	7,8355	0,664150735	1,107256999	3,42E-08	1,32E-07	lm.nb
CX3CR1	0,88421	0,27979	3,16029	0,335825595	1,432594466	0,0041	0,005895	lm.nb
GPI	0,8836088	0,11244	7,85823	0,663219093	1,103998512	3,25E-08	1,28E-07	lm.nb
TGFBR1	0,8809133	0,16547	5,32368	0,556590767	1,205235785	1,62E-05	3,25E-05	lm.nb
BST1	0,8805064	0,22069	3,98984	0,447960015	1,313052722	0,00051	0,000854	lm.nb
CD69	0,876281	0,42496	2,06203	0,043356717	1,709205282	0,04974	0,061366	lm.nb
IRAK1	0,8539455	0,09925	8,60385	0,659412357	1,048478662	6,08E-09	2,89E-08	lm.nb
BTLA	0,850351	0,28583	2,97505	0,290129771	1,410572322	0,00641	0,009027	lm.nb
BAX	0,848258	0,07968	10,6456	0,692081891	1,004434054	8,94E-11	9,18E-10	lm.nb
CCBP2	0,8435637	0,32095	2,6283	0,214494525	1,47263293	0,01446	0,019278	lm.nb
KLRB1	0,8410775	0,43698	1,92476	-0,015397181	1,697552166	0,06571	0,079415	lm.nb
CCL18	0,8379139	0,45338	1,84815	-0,050709575	1,726537426	0,07644	0,091652	lm.nb
IRF3	0,8330527	0,16446	5,06533	0,510707923	1,155397474	3,15E-05	6,05E-05	lm.nb
IKBKG	0,8178856	0,09125	8,96266	0,6390262	0,996744979	2,79E-09	1,51E-08	lm.nb
ATG7	0,817601	0,12378	6,60515	0,574987613	1,060214407	6,40E-07	1,73E-06	lm.nb
CASP1	0,7982965	0,16251	4,91227	0,479775439	1,116817606	4,68E-05	8,77E-05	lm.nb
XBP1	0,7894659	0,19291	4,09251	0,411371761	1,167559959	0,00039	0,000664	lm.nb
IL1RAP	0,7764976	0,24987	3,10764	0,286758338	1,266236776	0,00466	0,006617	lm.nb
LTBR	0,7655427	0,12477	6,13551	0,520988484	1,010096942	2,05E-06	4,91E-06	lm.nb
STAT6	0,755596	0,08512	8,87695	0,588763074	0,922428907	3,35E-09	1,74E-08	lm.nb
IRF4	0,7494308	0,22419	3,3428	0,310013047	1,188848636	0,00261	0,003885	lm.nb
IL13RA1	0,7486766	0,14192	5,27544	0,470518316	1,026834838	1,83E-05	3,66E-05	lm.nb
IKBKB	0,7401656	0,12375	5,98137	0,49762513	0,982706045	3,02E-06	7,05E-06	lm.nb
IFNAR1	0,731649	0,12324	5,9367	0,4900952	0,973202794	3,38E-06	7,85E-06	lm.nb
MAP4K4	0,7292989	0,13847	5,267	0,457906272	1,00069147	1,87E-05	3,69E-05	lm.nb
FAS	0,7266333	0,24469	2,96958	0,2470365	1,206230122	0,0065	0,009118	lm.nb
ITGA5	0,715048	0,19747	3,62096	0,327997164	1,102098863	0,0013	0,002016	lm.nb
BLNK	0,7131715	0,26426	2,69873	0,195217644	1,231125332	0,01229	0,016638	lm.nb
IGF2R	0,7074115	0,12908	5,48059	0,454423068	0,960400017	1,08E-05	2,26E-05	lm.nb
VEGFA	0,6989078	0,30431	2,29673	0,102468267	1,295347292	0,03029	0,03824	lm.nb
CARD14	0,6787102	0,24021	2,82546	0,20789333	1,14952708	0,00915	0,012604	lm.nb

PYCARD	0,6724418	0,18367	3,66122	0,312456785	1,032426718	0,00118	0,001833	lm.nb
ADA	0,6706499	0,22855	2,93436	0,222691031	1,118608723	0,00707	0,009857	lm.nb
EGR2	0,6699373	0,34371	1,94913	-0,003737481	1,343612141	0,06259	0,076044	lm.nb
CDKN1A	0,66738	0,27925	2,38988	0,120045179	1,214714881	0,02471	0,031642	lm.nb
CD276	0,6672679	0,17318	3,85297	0,327829901	1,006705856	0,00072	0,001179	lm.nb
LIF	0,6672018	0,3951	1,6887	-0,107188579	1,441592219	0,10371	0,121762	lm.nb
ENTPD1	0,6419939	0,21131	3,03817	0,227827817	1,056160013	0,00551	0,007804	lm.nb
NT5E	0,635474	0,23171	2,7425	0,181315657	1,089632321	0,01111	0,015167	lm.nb
CEBPB	0,6300173	0,13898	4,53305	0,357610278	0,902424368	0,00013	0,000222	lm.nb
MRC1	0,6243532	0,1822	3,42669	0,267235798	0,981470525	0,00212	0,003217	lm.nb
CD4	0,6204436	0,31187	1,98945	0,009185918	1,231701281	0,0577	0,070675	lm.nb
MCL1	0,6169651	0,22685	2,71972	0,172341173	1,061588965	0,01171	0,015895	lm.nb
IRAK4	0,6126386	0,12989	4,71643	0,358045349	0,86723194	7,77E-05	0,000142	lm.nb
TBK1	0,6008436	0,1061	5,66291	0,392884485	0,808802778	6,78E-06	1,49E-05	lm.nb
FCGR2B	0,6003459	0,24799	2,42088	0,114291721	1,086400083	0,02308	0,029801	lm.nb
TRAF4	0,5664802	0,18037	3,14063	0,212951931	0,920008497	0,0043	0,006165	lm.nb
SIGIRR	0,5624151	0,2172	2,58936	0,136698231	0,988131936	0,0158	0,020948	lm.nb
MBP	0,5618894	0,11174	5,02838	0,342872019	0,780906753	3,46E-05	6,55E-05	lm.nb
CFLAR_iso1	0,5583335	0,15042	3,7118	0,263507508	0,853159458	0,00103	0,001652	lm.nb
CD82	0,5454887	0,15262	3,57424	0,246359779	0,844617544	0,00146	0,002252	lm.nb
CD24	0,5279451	0,268	1,96992	0,002659304	1,053230836	0,06002	0,073125	lm.nb
MARCO	0,511507	0,41783	1,2242	-0,307436793	1,330450826	0,23229	0,263804	lm.nb
CHUK	0,4781185	0,08497	5,62706	0,311581706	0,644655349	7,43E-06	1,61E-05	lm.nb
ICOSLG	0,4705741	0,14581	3,22742	0,184795957	0,756352177	0,00347	0,005049	lm.nb
TIRAP	0,442615	0,13195	3,35454	0,184002633	0,701227377	0,00254	0,003786	lm.nb
IVL	0,4393749	0,3578	1,22798	-0,261919812	1,140669655	0,23089	0,262879	lm.nb
NOTCH1	0,4315237	0,11312	3,8146	0,209799934	0,653247435	0,0008	0,00129	lm.nb
C1QBP	0,4313154	0,11444	3,76903	0,207019438	0,655611374	0,00089	0,001444	lm.nb
PRDM1	0,4214769	0,22856	1,84409	-0,026493006	0,869446796	0,07705	0,092139	lm.nb
NFKBIZ	0,4015465	0,20076	2,00014	0,008058801	0,795034194	0,05646	0,069347	lm.nb
HLA_DPB1	0,3786592	0,30094	1,25824	-0,211189532	0,968507955	0,21993	0,251666	lm.nb
TFRC	0,3745076	0,13204	2,83622	0,115699727	0,633315495	0,00892	0,012326	lm.nb
LILRB5	0,3655492	0,28168	1,29773	-0,186549427	0,917647864	0,20623	0,238399	lm.nb
PTGER4	0,3550127	0,14173	2,50494	0,077231449	0,632794007	0,01913	0,02499	lm.nb
TNFAIP3	0,3241179	0,39686	0,81671	-0,453727631	1,101963334	0,42181	0,457215	lm.nb
MUC1	0,3174691	0,53688	0,59132	-0,734816506	1,369754712	0,55961	0,59798	lm.nb
HLA_DQB1	0,3123443	1,67374	0,18661	-2,968189151	3,592877792	0,85347	0,863016	loglinear
PDCD2	0,3036961	0,11307	2,68585	0,082074066	0,525318047	0,01267	0,017075	lm.nb
CXCL2	0,2984187	0,6355	0,46958	-0,947167351	1,544004703	0,64273	0,67561	lm.nb
PDGFB	0,2886364	0,17617	1,63838	-0,056661226	0,633934084	0,11387	0,13334	lm.nb
BCL6	0,2709869	0,12397	2,18593	0,028008116	0,513965659	0,0384	0,047945	lm.nb
S1PR1	0,2682787	0,26137	1,02643	-0,244005704	0,780563181	0,31452	0,351016	lm.nb
IFNGR1	0,259457	0,10146	2,55719	0,060591564	0,458322423	0,017	0,022471	lm.nb
RELA	0,2573426	0,09625	2,67381	0,068700915	0,445984192	0,01302	0,017467	lm.nb

BCL10	0,2519355	0,11637	2,16501	0,023856	0,480014987	0,04013	0,049836	lm.nb
TYK2	0,251691	0,10445	2,40962	0,046964253	0,456417754	0,02366	0,030466	lm.nb
MAF	0,2511206	0,17137	1,4654	-0,084758017	0,586999281	0,15528	0,180889	lm.nb
IKBKAP	0,2501141	0,13185	1,89697	-0,008310582	0,508538855	0,06944	0,083701	lm.nb
HLA_DOB	0,2496998	0,25446	0,9813	-0,249038462	0,748437997	0,33585	0,372066	lm.nb
TGFBI	0,2495349	0,19344	1,28997	-0,129611283	0,628681078	0,20887	0,240835	lm.nb
C1R	0,2418212	0,18889	1,28025	-0,128394722	0,612037035	0,21221	0,244067	lm.nb
CD8B	0,2370188	0,43532	0,54447	-0,616204181	1,090241813	0,59094	0,624077	lm.nb
MALT1	0,2363285	0,13545	1,74479	-0,029150536	0,501807552	0,09331	0,109828	lm.nb
CD59	0,1992433	0,1695	1,17551	-0,132967789	0,531454445	0,25087	0,283478	lm.nb
GPR183	0,1723407	0,30175	0,57113	-0,419097107	0,763778441	0,57301	0,609414	lm.nb
SLC2A1	0,1577641	0,27846	0,56656	-0,38801364	0,703541798	0,57606	0,611221	lm.nb
B2M	0,1558925	0,17574	0,88707	-0,188554085	0,500339178	0,38349	0,42175	lm.nb
STAT5A	0,1426505	0,17678	0,80695	-0,203833905	0,489134916	0,42731	0,462064	lm.nb
MAPK14	0,1332041	0,07247	1,83812	-0,008832167	0,275240346	0,07796	0,092972	lm.nb
IL31RA	0,1323155	0,54732	0,24175	-0,940434905	1,205065878	0,81095	0,825557	lm.nb
KLRC4	0,1216158	0,45479	0,26741	-0,769776059	1,013007723	0,79135	0,807424	lm.nb
CD58	0,1210521	0,1151	1,05167	-0,10455351	0,346657638	0,30301	0,339013	lm.nb
KLRK1	0,1203784	0,40829	0,29484	-0,679861582	0,920618371	0,77055	0,787985	lm.nb
CSF1	0,1077506	0,24105	0,447	-0,364708941	0,580210153	0,65872	0,690816	lm.nb
NFKB1	0,1069912	0,12481	0,85721	-0,137641927	0,351624316	0,39947	0,435086	lm.nb
MAP4K2	0,0969198	0,16788	0,57731	-0,232129292	0,425968812	0,56889	0,606463	lm.nb
ILF3	0,0890337	0,06065	1,46811	-0,029830787	0,207898098	0,15454	0,180502	lm.nb
UBE2L3	0,0850963	0,0893	0,95297	-0,089923755	0,260116449	0,34973	0,386498	lm.nb
TRAF6	0,0774642	0,0894	0,86648	-0,097762551	0,252690998	0,39447	0,431719	lm.nb
HLA_DQA1	0,067164	1,91446	0,03508	-3,685168151	3,819496081	0,97229	0,976614	loglinear
C5	0,0667451	0,18611	0,35864	-0,298024655	0,431514779	0,72288	0,747843	lm.nb
FCGRT	0,0586953	0,19295	0,3042	-0,319492099	0,436882707	0,7635	0,784319	lm.nb
FYN	0,0578972	0,16153	0,35844	-0,258695092	0,374489518	0,72303	0,747843	lm.nb
CCL13	0,037685	0,25222	0,14941	-0,456669967	0,53203988	0,88243	0,890306	lm.nb
CFLAR	0,0044133	0,1247	0,03539	-0,240000461	0,248827126	0,97205	0,976614	lm.nb
CCRL1	-1,37E-15	0,28323	-4,85E-15	-0,555135004	0,555135004	1	1	lm.nb
ITGA6	-0,003594	0,13388	-0,02684	-0,266003607	0,258815485	0,9788	0,980967	lm.nb
JAK1	-0,015053	0,07393	-0,2036	-0,159964361	0,129857971	0,84031	0,851617	lm.nb
PSMD7	-0,028551	0,06668	-0,42821	-0,159236014	0,102133186	0,67216	0,703282	lm.nb
TRAF5	-0,038767	0,1754	-0,22102	-0,382555421	0,305020899	0,82687	0,83988	lm.nb
PSMC2	-0,042808	0,06613	-0,64732	-0,17242328	0,08680786	0,52332	0,561858	lm.nb
AnnexinA1	-0,063582	0,20072	-0,31676	-0,457000915	0,32983771	0,75405	0,777139	lm.nb
ICAM2	-0,068037	0,21545	-0,31578	-0,490326898	0,354252975	0,75479	0,777139	lm.nb
CASP2	-0,074031	0,12516	-0,59149	-0,31934851	0,171285589	0,55951	0,59798	lm.nb
TNFRSF11A	-0,074882	0,19809	-0,37801	-0,463146118	0,313381329	0,70861	0,738	lm.nb
IL33	-0,082691	0,22704	-0,36421	-0,527689393	0,362308114	0,71876	0,746852	lm.nb
HFE	-0,095496	0,31848	-0,29984	-0,719725874	0,528733879	0,76677	0,7859	lm.nb
KLRG2	-0,099889	0,26115	-0,3825	-0,611744431	0,411965967	0,70533	0,736275	lm.nb

NFKBIA	-0,123595	0,25489	-0,4849	-0,623173267	0,375983286	0,63197	0,66585	lm.nb
TLR5	-0,126008	0,22607	-0,55738	-0,569110414	0,317094644	0,58223	0,616314	lm.nb
CRADD	-0,13022	0,10246	-1,27094	-0,331040171	0,070601116	0,21545	0,247167	lm.nb
HRAS	-0,141579	0,12105	-1,16957	-0,378842453	0,095683975	0,25321	0,285409	lm.nb
SMAD3	-0,150151	0,12964	-1,15823	-0,40424266	0,10394094	0,25772	0,289772	lm.nb
CD99	-0,150858	0,17565	-0,85885	-0,495130886	0,193415759	0,39858	0,435086	lm.nb
TGFBR2	-0,153835	0,17297	-0,88938	-0,492853359	0,185183634	0,38228	0,421437	lm.nb
PSMB5	-0,180644	0,10101	-1,78836	-0,378625104	0,017337506	0,08585	0,101311	lm.nb
IL16	-0,188246	0,15218	-1,23701	-0,486517366	0,110024622	0,22758	0,259762	lm.nb
XCR1	-0,193258	0,22978	-0,84104	-0,643633016	0,257118005	0,4083	0,443631	lm.nb
STAT5B	-0,196502	0,09961	-1,97268	-0,391741287	-0,001263283	0,05969	0,072916	lm.nb
DUSP4	-0,198944	0,22799	-0,87259	-0,645808891	0,247921101	0,39119	0,42917	lm.nb
CUL9	-0,200304	0,11037	-1,81477	-0,416638402	0,01602978	0,08158	0,096779	lm.nb
RAF1	-0,204995	0,10945	-1,87299	-0,41951427	0,009523271	0,07281	0,087526	lm.nb
IL36g	-0,216785	0,31943	-0,67867	-0,842862319	0,409291899	0,50358	0,541951	lm.nb
TNFSF12	-0,220934	0,20274	-1,08972	-0,618310068	0,17644234	0,28623	0,32103	lm.nb
CASP3	-0,233187	0,08968	-2,60027	-0,408955822	-0,057417922	0,01542	0,020494	lm.nb
ATG16L1	-0,254826	0,08466	-3,01016	-0,420750125	-0,088901736	0,00589	0,008323	lm.nb
TP53	-0,26072	0,09711	-2,68492	-0,451046072	-0,070393529	0,01269	0,017075	lm.nb
FKBP5	-0,264506	0,35508	-0,74492	-0,960462886	0,431451447	0,46327	0,499753	lm.nb
CFI	-0,271249	0,2289	-1,18504	-0,719884531	0,177386022	0,24715	0,279976	lm.nb
RIPK1	-0,272262	0,11722	-2,32259	-0,502020845	-0,042503968	0,02863	0,036356	lm.nb
PSMB7	-0,276291	0,08457	-3,26705	-0,442046341	-0,110536214	0,00315	0,004625	lm.nb
BCAP31	-0,279202	0,0891	-3,13353	-0,453841608	-0,104563238	0,00437	0,006253	lm.nb
ETS1	-0,282456	0,14666	-1,92598	-0,569902101	0,004989777	0,06555	0,079415	lm.nb
DPP4	-0,283646	0,28777	-0,98567	-0,847675613	0,280383681	0,33374	0,37064	lm.nb
NFATC1	-0,287168	0,1585	-1,81179	-0,597827362	0,023490612	0,08205	0,097084	lm.nb
SERPING1	-0,288391	0,21511	-1,34064	-0,710014746	0,13323253	0,19209	0,22263	lm.nb
NFATC3	-0,290072	0,0886	-3,27393	-0,463729241	-0,116414971	0,0031	0,004562	lm.nb
ATG5	-0,291748	0,06417	-4,54682	-0,417512694	-0,165984182	0,00012	0,000216	loglinear
NLRP1	-0,308019	0,14027	-2,19587	-0,582951215	-0,033086507	0,0376	0,047074	lm.nb
IL6ST	-0,337601	0,14025	-2,40714	-0,612489709	-0,06271156	0,02379	0,030546	lm.nb
CX3CL1	-0,411957	0,4047	-1,01792	-1,205176885	0,381262701	0,31847	0,354548	lm.nb
CTSG	-0,421419	0,30083	-1,40086	-1,01104296	0,168204571	0,17355	0,201653	lm.nb
ABL1	-0,42579	0,11486	-3,7071	-0,65091101	-0,200667994	0,00105	0,001666	lm.nb
IL32	-0,434245	0,23644	-1,83663	-0,897658338	0,029168048	0,07818	0,092996	lm.nb
ZEB1	-0,446493	0,18359	-2,43196	-0,806336802	-0,086648324	0,02252	0,029161	lm.nb
IL1RL2	-0,502303	0,20645	-2,4331	-0,906938068	-0,097668784	0,02246	0,029161	lm.nb
PTK2	-0,504422	0,07772	-6,49023	-0,656753977	-0,352090566	8,49E-07	2,22E-06	lm.nb
TCF4	-0,510028	0,15161	-3,364	-0,80719067	-0,212865769	0,00248	0,003711	lm.nb
CDH5	-0,511085	0,21829	-2,34133	-0,938930804	-0,083239055	0,02749	0,035098	lm.nb
TOLLIP	-0,511819	0,06518	-7,85229	-0,639572987	-0,384064337	3,29E-08	1,28E-07	lm.nb
BCL2	-0,515011	0,15854	-3,24843	-0,825752139	-0,204269496	0,0033	0,004811	lm.nb
C14orf166	-0,531809	0,08984	-5,91934	-0,707900632	-0,355717723	3,54E-06	8,16E-06	lm.nb

MR1	-0,550114	0,19197	-2,8657	-0,926365115	-0,173862015	0,00832	0,011532	lm.nb
NOTCH2	-0,553407	0,08102	-6,83062	-0,712202953	-0,39461027	3,69E-07	1,05E-06	lm.nb
CD46	-0,553887	0,09733	-5,69074	-0,744656027	-0,363117507	6,32E-06	1,39E-05	lm.nb
ATG10	-0,561736	0,15853	-3,54333	-0,872462166	-0,251010597	0,00158	0,002426	lm.nb
AHR	-0,564448	0,11866	-4,75678	-0,797025313	-0,331870898	7,00E-05	0,000128	lm.nb
MAPK1	-0,578074	0,0952	-6,07211	-0,764668595	-0,391478818	2,41E-06	5,67E-06	lm.nb
GP1BB	-0,609239	0,27363	-2,22648	-1,145558676	-0,072918442	0,03523	0,044351	lm.nb
CTNNB1	-0,620035	0,09	-6,8894	-0,796431448	-0,443637897	3,20E-07	9,27E-07	lm.nb
PECAM1	-0,627706	0,22917	-2,73908	-1,076871765	-0,178539362	0,0112	0,015242	lm.nb
TLR3	-0,648064	0,277	-2,33954	-1,190991749	-0,105135519	0,0276	0,035136	lm.nb
IL7	-0,671241	0,26683	-2,51562	-1,194226053	-0,148255349	0,01868	0,024468	lm.nb
APP	-0,671845	0,08025	-8,3724	-0,829125544	-0,514564433	1,01E-08	4,45E-08	lm.nb
CASP14	-0,683573	0,25879	-2,64143	-1,190799604	-0,176346398	0,01403	0,018762	lm.nb
NFIL3	-0,700271	0,28638	-2,44529	-1,261568411	-0,138974568	0,02186	0,028473	lm.nb
ABCB1	-0,704952	0,14418	-4,88952	-0,987537018	-0,422367104	4,96E-05	9,27E-05	lm.nb
C1S	-0,706878	0,19711	-3,58618	-1,093217616	-0,320539241	0,00142	0,002193	lm.nb
ITGB1	-0,726742	0,1286	-5,65125	-0,978794832	-0,474688772	6,99E-06	1,52E-05	lm.nb
IRF6	-0,75383	0,22116	-3,40856	-1,187298931	-0,320360416	0,00222	0,003354	lm.nb
CD1A	-0,76315	0,34501	-2,21197	-1,439369834	-0,086931087	0,03633	0,045618	lm.nb
ITGAE	-0,779351	0,10995	-7,08853	-0,994844121	-0,563858055	1,98E-07	5,89E-07	lm.nb
CD81	-0,781006	0,12346	-6,32616	-1,022980565	-0,539030816	1,28E-06	3,22E-06	lm.nb
IL36b	-0,799583	0,24787	-3,22585	-1,285404341	-0,313762604	0,00349	0,005053	lm.nb
IL36Ra	-0,807514	0,20959	-3,85278	-1,218315858	-0,396712146	0,00072	0,001179	lm.nb
NCAM1	-0,808056	0,1665	-4,85306	-1,134405179	-0,481707724	5,45E-05	0,000101	lm.nb
SKI	-0,831941	0,11448	-7,26693	-1,056328678	-0,607554267	1,29E-07	3,95E-07	lm.nb
TAL1	-0,833885	0,2528	-3,29864	-1,329366171	-0,338403879	0,00291	0,00432	lm.nb
LEF1	-0,84096	0,39753	-2,11547	-1,62011462	-0,061805091	0,04453	0,055139	lm.nb
PPARG	-0,846374	0,38963	-2,17225	-1,610048233	-0,0826996	0,03952	0,049215	lm.nb
CD164	-0,861804	0,10462	-8,23744	-1,066859858	-0,656748236	1,37E-08	5,91E-08	lm.nb
FN1	-0,879238	0,19173	-4,58573	-1,255034863	-0,503440216	0,00011	0,000196	lm.nb
NFATC2	-0,900238	0,2261	-3,98167	-1,343385566	-0,457090086	0,00052	0,000869	lm.nb
IL18	-0,948179	0,25672	-3,69339	-1,451355739	-0,445001869	0,00108	0,001713	lm.nb
CD9	-0,984961	0,16416	-5,99988	-1,306722077	-0,663200774	2,89E-06	6,76E-06	lm.nb
EDNRB	-1,024196	0,20362	-5,02986	-1,423297118	-0,625094999	3,45E-05	6,55E-05	lm.nb
PDGFRB	-1,072783	0,19722	-5,4394	-1,459342979	-0,686222451	1,20E-05	2,46E-05	lm.nb
SMAD5	-1,108316	0,10149	-10,9209	-1,307227754	-0,909404	5,26E-11	6,43E-10	lm.nb
LGALS3	-1,132548	0,15139	-7,48124	-1,429262445	-0,835833213	7,80E-08	2,54E-07	lm.nb
C3	-1,145174	0,2628	-4,35754	-1,660266727	-0,630080603	0,0002	0,000345	lm.nb
IL11RA	-1,18306	0,15815	-7,48051	-1,493039218	-0,873081589	7,81E-08	2,54E-07	lm.nb
CEACAM6	-1,207559	0,58584	-2,06123	-2,355812116	-0,059306522	0,04983	0,061366	lm.nb
RORC	-1,245559	0,20315	-6,13123	-1,643732626	-0,84738464	2,07E-06	4,94E-06	lm.nb
GATA3	-1,370856	0,26225	-5,22728	-1,884866028	-0,856845481	2,07E-05	4,07E-05	lm.nb
CXCL12	-1,394763	0,21903	-6,36805	-1,824051975	-0,965473434	1,15E-06	2,92E-06	lm.nb
EGR1	-1,404038	0,38267	-3,66903	-2,15407752	-0,653999094	0,00115	0,001803	lm.nb

IKZF2	-1,442333	0,20148	-7,15868	-1,837234718	-1,047432139	1,67E-07	5,01E-07	lm.nb
CD36	-1,512049	0,39158	-3,86137	-2,27955292	-0,744544629	0,00071	0,001162	lm.nb
CFH	-1,712972	0,20329	-8,42627	-2,111418908	-1,314524467	9,00E-09	4,03E-08	lm.nb
CFD	-1,857823	0,31954	-5,81405	-2,484121996	-1,23152342	4,62E-06	1,04E-05	lm.nb
C7	-2,132558	0,4375	-4,87437	-2,990066556	-1,275049742	5,16E-05	9,60E-05	lm.nb
KIT	-2,202753	0,23005	-9,57524	-2,65364473	-1,751861049	7,64E-10	5,11E-09	lm.nb
FCER1A	-2,235901	0,3567	-6,26833	-2,935029741	-1,536773036	1,47E-06	3,68E-06	lm.nb
CD34	-2,287215	0,32676	-6,99959	-2,927672474	-1,64675764	2,45E-07	7,15E-07	lm.nb
ARG1	-2,315814	0,37627	-6,1547	-3,053298069	-1,578329578	1,96E-06	4,70E-06	lm.nb
CD55	-2,342815	0,30734	-7,62289	-2,945201134	-1,740429788	5,60E-08	1,95E-07	lm.nb
ZBTB16	-2,796615	0,47691	-5,86407	-3,731351785	-1,861878094	4,07E-06	9,28E-06	lm.nb
FLG2	-3,139023	0,39454	-7,9562	-3,912317729	-2,365729049	2,60E-08	1,04E-07	lm.nb
LOR	-3,278246	0,44397	-7,38398	-4,148422923	-2,408069688	9,81E-08	3,08E-07	lm.nb
FLG1	-3,607611	0,41799	-8,6308	-4,426876482	-2,788344848	5,73E-09	2,78E-08	lm.nb
