# Supplementary material for: Type I and II Interferon Signalling Characterizes the Transcriptional Landscape of Sweet Syndrome
Source: Exp Dermatol. 2026 Jul 6;35(7):e70323. doi: 10.1111/exd.70323 (PMC13338580; doi:10.1111/exd.70323)
Supplement: Supplementary file 2 — Table S2: Sweet syndrome–specific transcriptional signatures in comparison with pyoderma gangrenosum and healthy controls. Table summarizing differential gene expression analyses comparing SS with PG and HC skin samples. The table highlights a subset of genes selectively upregulated in SS, including interferon‐related genes that distinguish SS from both PG and healthy controls. [file EXD-35-e70323-s004.pdf]

Suppl.Table 2. Sweet syndrome–specific transcriptional signatures in comparison with pyoderma gangrenosum and healthy controls.								
	Log2FC	StdError	stat	LowerConfLimit	UpperConfLimit	pvalue	pvalue.adj	method
CXCL11	3,1706593	1,5398137	2,0591	0,152624544	6,188694145	0,05346	0,1940277	loglinear
CCL8	2,9837272	0,8811395	3,3862	1,256693886	4,710760556	0,0031	0,0454474	lm.nb
IFIT2	2,8221067	0,7298952	3,8665	1,391512038	4,25270134	0,00104	0,0289202	lm.nb
PLA2G2A	2,3491271	0,657663	3,5719	1,060107681	3,638146602	0,00203	0,0398714	lm.nb
MX1	2,3363268	0,5878813	3,9741	1,184079401	3,488574111	0,00081	0,0248921	lm.nb
CXCL10	2,104171	1,5621228	1,347	-0,9575898	5,165931764	0,19383	0,4111532	loglinear
OAS1	2,0695257	0,4863279	4,2554	1,116322981	3,022728414	0,00043	0,0190523	lm.nb
GBP1	1,9334604	0,5929449	3,2608	0,771288453	3,09563237	0,00411	0,0499501	lm.nb
GBP5	1,893546	0,6481455	2,9215	0,623180751	3,163911266	0,00876	0,069924	lm.nb
CCL24	1,7026217	0,6833082	2,4917	0,363337712	3,041905753	0,02212	0,1129164	lm.nb
CCL19	1,6959451	0,4772839	3,5533	0,760468703	2,631421529	0,00212	0,0399941	lm.nb
FCGR1AB	1,6839725	0,5459333	3,0846	0,613943236	2,754001778	0,0061	0,0551867	lm.nb
IRF7	1,6353752	0,4428761	3,6926	0,767338022	2,503412308	0,00155	0,0359923	lm.nb
CCL2	1,6267079	0,515391	3,1563	0,616541588	2,63687422	0,0052	0,0519925	lm.nb
IFI35	1,6104044	0,3942593	4,0846	0,837656091	2,383152677	0,00063	0,0238036	lm.nb
RARRES3	1,5930672	0,5302724	3,0042	0,553733204	2,632401162	0,00729	0,0616123	lm.nb
SELE	1,5285403	0,4719115	3,239	0,603593715	2,453486854	0,00432	0,0499501	lm.nb
CXCL9	1,5020184	1,2333214	1,2179	-0,915291569	3,919328466	0,23818	0,4802891	loglinear
CFD	1,3987672	0,4533107	3,0857	0,510278328	2,287256075	0,00609	0,0551867	lm.nb
IFIH1	1,3826692	0,3932997	3,5156	0,611801789	2,153536709	0,00231	0,0412599	lm.nb
IRF1	1,370439	0,438376	3,1262	0,511221976	2,22965596	0,00556	0,0538662	lm.nb
IDO1	1,3511472	1,3846074	0,9758	-1,362683365	4,064977769	0,34141	0,5808683	loglinear
CD274	1,3428792	0,5933763	2,2631	0,179861749	2,505896679	0,03553	0,1475485	lm.nb
GATA3	1,3325232	0,3454898	3,8569	0,655363294	2,009683127	0,00106	0,0289202	lm.nb
BATF3	1,2668619	0,3971005	3,1903	0,488544893	2,045178826	0,00482	0,0507814	lm.nb
TAP1	1,2426586	0,3424873	3,6283	0,571383547	1,913933637	0,00179	0,038119	lm.nb
GZMB	1,1555059	0,3585135	3,223	0,452819471	1,858192244	0,00448	0,0499501	lm.nb
LILRA1	1,127162	0,4359804	2,5854	0,272640356	1,981683579	0,01814	0,099998	lm.nb
KLRG2	1,1086571	0,3560457	3,1138	0,410807475	1,806506652	0,00572	0,0538662	lm.nb
TAP2	1,0873103	0,3199935	3,3979	0,460123048	1,714497649	0,00302	0,0454474	lm.nb
CX3CL1	1,074738	0,5334597	2,0147	0,029156918	2,120319016	0,05832	0,2003247	lm.nb
CIITA	1,0549357	0,413325	2,5523	0,244818754	1,865052596	0,01946	0,1048019	lm.nb
STAT1	1,0509664	0,4153216	2,5305	0,236936037	1,864996843	0,02038	0,10857	lm.nb
CFP	0,9888738	0,3400665	2,9079	0,322343372	1,655404152	0,00902	0,069924	lm.nb
IRF5	0,986264	0,303439	3,2503	0,391523581	1,581004482	0,00421	0,0499501	lm.nb
PML	0,9655519	0,3313176	2,9143	0,316169305	1,614934456	0,0089	0,069924	lm.nb
LILRB5	0,9603974	0,3911469	2,4553	0,193749421	1,727045315	0,02388	0,1146492	lm.nb
TNFSF10	0,9426598	0,337734	2,7911	0,280701067	1,604618438	0,01165	0,0796067	lm.nb
LILRA2	0,9395189	0,4775274	1,9675	0,003565225	1,875472568	0,0639	0,2060017	lm.nb

PSMB10	0,9352712	0,3079035	3,0375	0,331780394	1,538762044	0,00677	0,0582303	lm.nb
HAVCR2	0,9117556	0,2962395	3,0778	0,331126182	1,492385093	0,00619	0,0551867	lm.nb
LILRA6	0,9108595	0,4415059	2,0631	0,045507981	1,776211015	0,05304	0,1940277	lm.nb
EGR2	0,9081317	0,4935582	1,84	-0,059242338	1,875505697	0,08145	0,240423	lm.nb
CCL13	0,8523916	0,3737499	2,2806	0,119841752	1,584941445	0,03428	0,1435812	lm.nb
LILRB2	0,8496836	0,3186612	2,6664	0,22510756	1,474259644	0,01525	0,0934275	lm.nb
LTB4R	0,8150484	0,3008303	2,7093	0,225420922	1,404675851	0,01391	0,087361	lm.nb
PSMB9	0,7987553	0,3464056	2,3058	0,119800274	1,477710359	0,03256	0,1399531	lm.nb
SOCS1	0,7954758	0,5131318	1,5502	-0,210262435	1,801214098	0,13758	0,3288538	lm.nb
CASP1	0,7843218	0,2299029	3,4115	0,333712188	1,234931401	0,00293	0,0454474	lm.nb
IL10	0,7759387	0,3936587	1,9711	0,004367635	1,547509678	0,06346	0,2060017	lm.nb
CCRL1	0,7739683	0,3462995	2,235	0,095221178	1,452715343	0,03762	0,1524062	lm.nb
TNFSF13B	0,7643944	0,3796596	2,0134	0,020261621	1,508527179	0,05846	0,2003247	lm.nb
HLA_DRB1	0,742882	0,8274627	0,8978	-0,878944868	2,364708864	0,38054	0,6039118	lm.nb
IL12RB1	0,7381683	0,3691018	1,9999	0,014728758	1,461607925	0,06001	0,2039645	lm.nb
CD3EAP	0,7195143	0,3374585	2,1322	0,0580956	1,380932966	0,04626	0,1784434	lm.nb
PSMB8	0,7175928	0,2206396	3,2523	0,285139208	1,150046343	0,00419	0,0499501	lm.nb
LTB4R2	0,714563	0,2879433	2,4816	0,15019416	1,278931935	0,0226	0,1133788	lm.nb
JAK2	0,6927789	0,2606334	2,6581	0,181937457	1,203620382	0,01553	0,0939461	lm.nb
TNFSF11	0,6851349	0,4327388	1,5833	-0,163033222	1,533302994	0,12987	0,3213893	lm.nb
CCR5	0,6818222	0,3724334	1,8307	-0,048147282	1,411791749	0,08287	0,2431603	lm.nb
C5	0,6775788	0,229668	2,9503	0,227429396	1,127728124	0,00822	0,067561	lm.nb
FLG2	0,6753888	0,7697849	0,8774	-0,833389607	2,184167196	0,39124	0,6098215	lm.nb
CCR1	0,6710066	0,3426594	1,9582	-0,000605874	1,342619139	0,06505	0,2083305	lm.nb
CD9	0,6657131	0,2342267	2,8422	0,206628641	1,124797462	0,01042	0,0785441	lm.nb
MUC1	0,6545884	0,4238165	1,5445	-0,176092062	1,485268771	0,13896	0,3299426	lm.nb
LILRA3	0,6332891	0,2662131	2,3789	0,111511461	1,155066691	0,02801	0,1270684	lm.nb
RORC	0,6202206	0,3335598	1,8594	-0,033556588	1,273997849	0,07853	0,2332036	lm.nb
IL1RL2	0,6185709	0,3651779	1,6939	-0,097177839	1,334319625	0,10662	0,2914964	lm.nb
CD86	0,6014784	0,4068458	1,4784	-0,195939433	1,398896265	0,15569	0,3548276	lm.nb
GP1BB	0,6010731	0,3976947	1,5114	-0,178408579	1,38055482	0,14714	0,3433321	lm.nb
CTSS	0,5970271	0,3386307	1,7631	-0,066689141	1,260743294	0,09397	0,2676972	lm.nb
LILRB3	0,5908565	0,3705978	1,5943	-0,135515212	1,317228151	0,12736	0,3184027	lm.nb
MAF	0,5821285	0,2251346	2,5857	0,14086462	1,023392433	0,01813	0,099998	lm.nb
CD40	0,56969	0,2914498	1,9547	-0,001551586	1,140931599	0,0655	0,2083991	lm.nb
STAT2	0,5375981	0,2869353	1,8736	-0,024795051	1,099991284	0,07645	0,2284262	lm.nb
IL15	0,5267335	0,3604606	1,4613	-0,179769289	1,233236329	0,16028	0,3602638	lm.nb
LILRB1	0,5261803	0,5263861	0,9996	-0,505536506	1,55789701	0,33006	0,5766497	lm.nb
IRF6	0,5227669	0,3251648	1,6077	-0,114556047	1,160089841	0,12439	0,315616	lm.nb
HRAS	0,522284	0,1906241	2,7399	0,148660697	0,895907332	0,01302	0,0828406	lm.nb
NOD1	0,5182476	0,186434	2,7798	0,152836993	0,883658139	0,01194	0,0801271	lm.nb
CCR2	0,5116607	0,3697394	1,3838	-0,213028637	1,236349983	0,18245	0,3926619	lm.nb

LILRA5	0,5114603	0,5643963	0,9062	-0,594756412	1,617676982	0,37618	0,6039118	lm.nb
CARD9	0,5105283	0,3584813	1,4241	-0,192095077	1,213151761	0,17062	0,3783033	lm.nb
CCBP2	0,504967	0,4889521	1,0328	-0,453379127	1,463313155	0,31469	0,5607145	lm.nb
TICAM1	0,502371	0,245424	2,047	0,021340044	0,983402024	0,05475	0,1972562	lm.nb
HLA_DMB	0,5004909	0,3598279	1,3909	-0,204771671	1,205753555	0,18033	0,3926619	lm.nb
CSF1R	0,4982471	0,3450382	1,444	-0,178027809	1,174521983	0,16502	0,3692173	lm.nb
CYBB	0,4924305	0,3340952	1,4739	-0,162396138	1,147257044	0,15688	0,3558819	lm.nb
MLKL	0,4798805	0,2963892	1,6191	-0,101042378	1,060803287	0,12191	0,3111197	lm.nb
LOR	0,4773123	0,8050274	0,5929	-1,100541428	2,055166069	0,56023	0,7320342	lm.nb
IL32	0,4771297	0,1993975	2,3929	0,086310661	0,867948682	0,02721	0,1245884	lm.nb
STAT6	0,4737588	0,1086211	4,3616	0,260861396	0,686656118	0,00034	0,0164558	lm.nb
IFITM1	0,4670038	0,2970869	1,5719	-0,115286601	1,049294131	0,13247	0,324547	lm.nb
CD1A	0,4627552	0,5569418	0,8309	-0,628850701	1,554361029	0,41636	0,6282415	lm.nb
CD80	0,4611639	0,4644226	0,993	-0,449104466	1,37143225	0,3332	0,577137	lm.nb
PRF1	0,453672	0,3680685	1,2326	-0,267742299	1,175086272	0,23277	0,4732651	lm.nb
GNLY	0,4309127	0,599429	0,7189	-0,743968162	1,605793498	0,48097	0,6772254	lm.nb
NCAM1	0,4283502	0,2693231	1,5905	-0,09952319	0,956223498	0,12823	0,3189489	lm.nb
CD209	0,4257172	0,3523063	1,2084	-0,264803109	1,116237504	0,24173	0,4838576	lm.nb
IFI16	0,4064469	0,1544771	2,6311	0,103671703	0,709222068	0,01645	0,0983209	lm.nb
MYD88	0,4050589	0,2453406	1,651	-0,075808564	0,885926428	0,11517	0,3017815	lm.nb
C1QBP	0,4044425	0,1442786	2,8032	0,121656323	0,687228599	0,01134	0,0796067	lm.nb
LGALS3	0,4038937	0,1841126	2,1937	0,043032985	0,764754426	0,04089	0,1615988	lm.nb
CMKLR1	0,3923244	0,3549484	1,1053	-0,303374351	1,088023247	0,28283	0,5309834	lm.nb
HLA_DMA	0,3906617	0,3425354	1,1405	-0,280707682	1,062031148	0,26825	0,5154624	lm.nb
CD8A	0,3881872	0,503096	0,7716	-0,597881075	1,374255422	0,44984	0,6446022	lm.nb
AIM2	0,3875609	0,4723461	0,8205	-0,538237529	1,313359298	0,42211	0,6305842	lm.nb
CFB	0,3820728	0,3868062	0,9878	-0,376067414	1,140213029	0,33568	0,577137	lm.nb
CD97	0,3752883	0,2652374	1,4149	-0,144576953	0,895153491	0,17327	0,3824522	lm.nb
PTAFR	0,3710383	0,3071914	1,2078	-0,231056844	0,973133457	0,24193	0,4838576	lm.nb
IRF3	0,3645745	0,2178688	1,6734	-0,062448238	0,791597325	0,11064	0,2934445	lm.nb
PTPN6	0,3628812	0,1283409	2,8275	0,111333139	0,614429273	0,01076	0,0786385	lm.nb
KLRC2	0,3601152	0,5823512	0,6184	-0,781293192	1,501523522	0,54367	0,7204479	lm.nb
BST2	0,3589873	0,4238856	0,8469	-0,471828531	1,189803111	0,40759	0,6236008	lm.nb
CLEC7A	0,3577779	0,3613795	0,99	-0,350525823	1,066081705	0,3346	0,577137	lm.nb
ATG12	0,3570319	0,2756587	1,2952	-0,183259128	0,897322919	0,21077	0,4357692	lm.nb
CD1D	0,3569467	0,3978399	0,8972	-0,422819446	1,136712814	0,38083	0,6039118	lm.nb
FASL	0,350783	0,3475874	1,0092	-0,330488278	1,032054193	0,32556	0,5717757	lm.nb
PYCARD	0,347103	0,216639	1,6022	-0,077509337	0,771715431	0,1256	0,315616	lm.nb
TLR7	0,345921	0,5779746	0,5985	-0,786909243	1,478751144	0,55657	0,7291977	lm.nb
TMEM173	0,345716	0,1740413	1,9864	0,004595106	0,686836897	0,06161	0,2039645	lm.nb
NT5E	0,3433017	0,1910276	1,7971	-0,031112376	0,717715867	0,08823	0,2528207	lm.nb
C1QB	0,3409881	0,5244593	0,6502	-0,686952092	1,368928216	0,52337	0,71179	lm.nb

ITGAX	0,3379684	0,4357802	0,7755	-0,516160823	1,192097651	0,44756	0,6446022	lm.nb
MBP	0,3356021	0,1504345	2,2309	0,04075049	0,63045366	0,03794	0,1524062	lm.nb
TLR8	0,3278301	0,3527014	0,9295	-0,363464676	1,019124908	0,3643	0,5990136	lm.nb
TRAF2	0,3243182	0,1290895	2,5124	0,071302852	0,577333528	0,02118	0,1092503	lm.nb
CDKN1A	0,3208083	0,338731	0,9471	-0,343104517	0,984721194	0,35548	0,5884707	lm.nb
IKBKAP	0,3193874	0,1840699	1,7351	-0,041389674	0,68016446	0,09891	0,2792114	lm.nb
BCL3	0,3192615	0,3366179	0,9484	-0,340509492	0,979032548	0,35481	0,5884707	lm.nb
CARD14	0,317197	0,359612	0,8821	-0,387642554	1,022036527	0,38877	0,6098215	lm.nb
CD7	0,3151672	0,5262752	0,5989	-0,71633209	1,34666651	0,55634	0,7291977	lm.nb
FCER1G	0,3126603	0,3847471	0,8126	-0,441444137	1,066764652	0,42649	0,6310126	lm.nb
MIF	0,3079998	0,187566	1,6421	-0,059629587	0,675629236	0,11702	0,3033855	lm.nb
IKBKG	0,3012121	0,0966072	3,1179	0,111862057	0,490562179	0,00566	0,0538662	lm.nb
TAPBP	0,2963188	0,196911	1,5048	-0,089626778	0,682264373	0,14881	0,3447197	lm.nb
LAMP3	0,294734	0,4321929	0,682	-0,552364082	1,141832021	0,5035	0,6958404	lm.nb
PSMC2	0,2942851	0,0863349	3,4086	0,125068682	0,463501519	0,00295	0,0454474	lm.nb
UBE2L3	0,2938808	0,1200806	2,4474	0,058522819	0,529238862	0,02428	0,1146492	lm.nb
TNFRSF11A	0,2794015	0,2445042	1,1427	-0,199826786	0,75862987	0,26735	0,5154624	lm.nb
IKBKB	0,278402	0,1181962	2,3554	0,046737512	0,510066429	0,0294	0,1301938	lm.nb
HLA_A	0,2740958	0,2365672	1,1586	-0,189575892	0,737767427	0,26096	0,5054161	lm.nb
ICOSLG	0,2740226	0,1980953	1,3833	-0,114244096	0,662289382	0,18262	0,3926619	lm.nb
GPI	0,2676776	0,125219	2,1377	0,022248379	0,513106801	0,04576	0,1779429	lm.nb
LTBR	0,2628691	0,1773294	1,4824	-0,084696525	0,610434747	0,15464	0,3548276	lm.nb
CD59	0,2557527	0,2168085	1,1796	-0,169192001	0,680697415	0,25271	0,4953106	lm.nb
GZMA	0,2554743	0,4759886	0,5367	-0,677463351	1,188411996	0,59769	0,7528716	lm.nb
XCR1	0,2505038	0,2891612	0,8663	-0,316252279	0,817259819	0,39713	0,613636	lm.nb
TP53	0,245097	0,1350581	1,8148	-0,019616951	0,509810926	0,08538	0,249034	lm.nb
PSMB7	0,2439068	0,098563	2,4746	0,050723404	0,437090283	0,02293	0,1135058	lm.nb
CCRL2	0,2407213	0,6435911	0,374	-1,02071718	1,5021598	0,71252	0,8293041	lm.nb
MASP1	0,2396811	0,3106731	0,7715	-0,369238275	0,848600428	0,44991	0,6446022	lm.nb
ITGA6	0,2396541	0,1999221	1,1987	-0,152193284	0,631501468	0,24537	0,4867629	lm.nb
IL6ST	0,2391613	0,1414764	1,6905	-0,03813238	0,516455001	0,10728	0,2914964	lm.nb
IL36b	0,2340553	0,3023346	0,7742	-0,358520525	0,826631202	0,44836	0,6446022	lm.nb
CCND3	0,2181102	0,2130102	1,0239	-0,199389684	0,63561017	0,31872	0,5638064	lm.nb
ATG10	0,2173165	0,1622097	1,3397	-0,100614586	0,535247492	0,19614	0,4142604	lm.nb
TLR4	0,2164424	0,2685047	0,8061	-0,309826723	0,742711508	0,43016	0,6329651	lm.nb
CD44	0,2095904	0,2585112	0,8108	-0,297091579	0,716272474	0,42754	0,6310126	lm.nb
IL18	0,2085562	0,3338154	0,6248	-0,445721902	0,86283437	0,53956	0,7204479	lm.nb
CTSC	0,2080549	0,1768075	1,1767	-0,138487698	0,554597587	0,25384	0,4955345	lm.nb
MSR1	0,2042246	0,3670749	0,5564	-0,515242263	0,923691508	0,58446	0,7485054	lm.nb
CLEC4E	0,2002851	0,5441898	0,368	-0,866326834	1,266897061	0,71691	0,8324307	lm.nb
SELPLG	0,1900711	0,2518632	0,7547	-0,303580785	0,683723054	0,45971	0,6567219	lm.nb
VCAM1	0,1871288	0,3211698	0,5826	-0,442364061	0,816621621	0,56698	0,7349765	lm.nb

CD163	0,1848476	0,3405461	0,5428	-0,482622787	0,85231796	0,59358	0,7515614	lm.nb
SLAMF7	0,1836407	0,5227748	0,3513	-0,840997928	1,208279349	0,72924	0,8340041	lm.nb
TGFBR2	0,1822908	0,1609247	1,1328	-0,133121641	0,497703261	0,2714	0,5174621	lm.nb
TGFB1	0,176267	0,2110695	0,8351	-0,23742921	0,589963115	0,41403	0,6280982	lm.nb
BATF	0,1751075	0,3318049	0,5277	-0,475230027	0,82544503	0,60379	0,7559903	lm.nb
CD53	0,1735819	0,2542201	0,6828	-0,324689486	0,671853378	0,50297	0,6958404	lm.nb
PSMB5	0,1714798	0,0862492	1,9882	0,002431317	0,340528186	0,06139	0,2039645	lm.nb
STAT4	0,1651627	0,4869354	0,3392	-0,789230805	1,119556143	0,73819	0,8340041	lm.nb
PDCD2	0,1645894	0,1527094	1,0778	-0,134720972	0,463899751	0,29462	0,5377902	lm.nb
STAT5A	0,1614002	0,1526333	1,0574	-0,137760994	0,460561477	0,30357	0,5469679	lm.nb
LAIR1	0,1608839	0,2827522	0,569	-0,393310416	0,715078214	0,57603	0,7427707	lm.nb
PTPN2	0,1581151	0,1694737	0,933	-0,174053414	0,490283596	0,36254	0,5981257	lm.nb
IL10RA	0,1559931	0,2648913	0,5889	-0,363193883	0,675180021	0,56287	0,7335256	lm.nb
ARHGDIB	0,1551575	0,1739571	0,8919	-0,185798433	0,496113347	0,38359	0,6063161	lm.nb
MCL1	0,1548449	0,2116987	0,7314	-0,260084504	0,569774246	0,47344	0,6724161	lm.nb
PSMD7	0,1538461	0,0762597	2,0174	0,004377027	0,303315245	0,05801	0,2003247	lm.nb
HLA_DPA1	0,1529239	0,4423422	0,3457	-0,71406681	1,019914605	0,73336	0,8340041	lm.nb
ATG16L1	0,1521945	0,0886261	1,7173	-0,02151255	0,325901643	0,10219	0,2861245	lm.nb
MRC1	0,1519314	0,3410818	0,4454	-0,516588896	0,820451664	0,66104	0,7982297	lm.nb
ICAM1	0,1507959	0,3989454	0,378	-0,631137048	0,932728752	0,70963	0,8293041	lm.nb
TLR5	0,1488007	0,2949072	0,5046	-0,429217369	0,726818692	0,61966	0,7675327	lm.nb
CD74	0,1464602	0,3683875	0,3976	-0,575579212	0,868499666	0,69538	0,8230314	lm.nb
SERPING1	0,1446751	0,2900815	0,4987	-0,423884749	0,713234863	0,62369	0,7678569	lm.nb
FKBP5	0,1399662	0,364716	0,3838	-0,574877177	0,854809528	0,70541	0,8289008	lm.nb
SRC	0,1395744	0,2229666	0,626	-0,297440133	0,576589031	0,53877	0,7204479	lm.nb
FCGRT	0,1351304	0,2461542	0,549	-0,347331844	0,617592694	0,58942	0,7485054	lm.nb
BST1	0,1331368	0,3657339	0,364	-0,58370164	0,849975265	0,71986	0,8338778	lm.nb
RIPK3	0,1304858	0,148219	0,8804	-0,160023508	0,420995036	0,38967	0,6098215	lm.nb
CUL9	0,1285512	0,1186184	1,0837	-0,103940897	0,361043368	0,29204	0,5377902	lm.nb
C1QA	0,124015	0,5088816	0,2437	-0,87339288	1,121422976	0,81007	0,8860187	lm.nb
RAF1	0,1228114	0,1350128	0,9096	-0,14181361	0,387436372	0,37441	0,6039118	lm.nb
MAPK1	0,1164852	0,1365478	0,8531	-0,151148499	0,384118943	0,40425	0,6209411	lm.nb
RELB	0,1163709	0,2406995	0,4835	-0,355400167	0,588141933	0,63429	0,7731361	lm.nb
CD36	0,1148796	0,4607972	0,2493	-0,788282853	1,018041983	0,8058	0,8833146	lm.nb
IKBKE	0,1139473	0,2076888	0,5486	-0,293122654	0,521017331	0,58964	0,7485054	lm.nb
TOLLIP	0,108412	0,0986214	1,0993	-0,084885903	0,301709969	0,28538	0,5337307	lm.nb
SMAD3	0,1077381	0,1866798	0,5771	-0,258154223	0,473630521	0,57063	0,7377522	lm.nb
ITGB2	0,1058466	0,31411	0,337	-0,509809021	0,721502123	0,73983	0,8340041	lm.nb
NFKB1	0,1039578	0,3091717	0,3362	-0,502018656	0,709934337	0,74037	0,8340041	lm.nb
HLA_DRB3	0,1011778	0,3448394	0,2934	-0,574707426	0,777063042	0,77239	0,8582143	lm.nb
C14orf166	0,0980054	0,1075867	0,9109	-0,112864623	0,308875331	0,37374	0,6039118	lm.nb
CTSG	0,0978532	0,3761734	0,2601	-0,639446652	0,835153112	0,79756	0,8762465	lm.nb

NOD2	0,0940118	0,2869587	0,3276	-0,468427142	0,656450793	0,74679	0,836188	lm.nb
TRAF4	0,0874954	0,1822843	0,48	-0,269781879	0,444772666	0,63671	0,7741648	lm.nb
FAS	0,087074	0,2557866	0,3404	-0,414267742	0,588415686	0,73728	0,8340041	lm.nb
NOTCH1	0,0863254	0,1320611	0,6537	-0,172514314	0,345165145	0,52115	0,71179	lm.nb
IL6R	0,0857065	0,1971805	0,4347	-0,300767347	0,472180388	0,66871	0,8050757	lm.nb
CD45RB	0,0844758	0,2377784	0,3553	-0,381569835	0,550521463	0,7263	0,8340041	lm.nb
STAT3	0,0767458	0,1853819	0,414	-0,286602675	0,440094358	0,68352	0,812923	lm.nb
NOTCH2	0,0737472	0,1193578	0,6179	-0,160194016	0,307688452	0,544	0,7204479	lm.nb
ILF3	0,0724876	0,0737915	0,9823	-0,072143755	0,217118911	0,33828	0,5775541	lm.nb
PPARG	0,0694258	0,3640169	0,1907	-0,644047312	0,782898872	0,85077	0,912201	lm.nb
TNFRSF14	0,063884	0,1889539	0,3381	-0,306465594	0,434233665	0,739	0,8340041	lm.nb
FCGR3AB	0,0597757	0,3945932	0,1515	-0,713626987	0,833178311	0,88119	0,9297781	lm.nb
CD68	0,0581031	0,3146307	0,1847	-0,558572998	0,674779199	0,85544	0,9152131	lm.nb
CASP14	0,0577088	0,4484181	0,1287	-0,821190747	0,936608298	0,89895	0,9332343	lm.nb
CD45R0	0,057303	0,2495323	0,2296	-0,431780381	0,54638631	0,82082	0,8937866	lm.nb
IL33	0,0540315	0,3277454	0,1649	-0,588349417	0,696412393	0,8708	0,9256795	lm.nb
CISH	0,0537209	0,3550993	0,1513	-0,642273743	0,749715502	0,88135	0,9297781	lm.nb
IFNAR2	0,0506293	0,1933049	0,2619	-0,328248407	0,429506949	0,79621	0,8762465	lm.nb
SMAD5	0,049991	0,1630394	0,3066	-0,269566315	0,369548265	0,76247	0,8491124	lm.nb
HLA_DRA	0,0438908	0,3864632	0,1136	-0,713577123	0,801358709	0,91077	0,9404131	lm.nb
FCGR2AC	0,0347846	0,3617396	0,0962	-0,674224943	0,743794195	0,9244	0,9436594	lm.nb
IRAK1	0,0340491	0,0948786	0,3589	-0,151912971	0,220011263	0,72365	0,8340041	lm.nb
HLA_C	0,0273593	0,2713639	0,1008	-0,504514037	0,559232571	0,92075	0,9431137	lm.nb
IGF2R	0,0269115	0,1948394	0,1381	-0,354973727	0,408796719	0,8916	0,9297781	lm.nb
NFKB2	0,0252822	0,2350267	0,1076	-0,435370122	0,485934519	0,91546	0,9404131	lm.nb
RELA	0,0240539	0,1107186	0,2173	-0,192954601	0,241062354	0,83033	0,90167	lm.nb
ZBTB16	0,0217158	0,5780244	0,0376	-1,111211909	1,15464358	0,97042	0,9804276	lm.nb
TBK1	0,0204683	0,149759	0,1367	-0,273059286	0,313995907	0,89273	0,9297781	lm.nb
CD81	0,0183846	0,1357872	0,1354	-0,247758329	0,284527547	0,89373	0,9297781	lm.nb
CD83	0,0170064	0,4274216	0,0398	-0,820739869	0,85475272	0,96868	0,9804276	lm.nb
FADD	0,0059493	0,2241824	0,0265	-0,433448142	0,445346675	0,97911	0,9857907	lm.nb
MALT1	0,0034609	0,1659862	0,0209	-0,321872111	0,3287939	0,98358	0,9857907	lm.nb
TLR1	-0,005136	0,2277374	-0,0226	-0,451501281	0,441229323	0,98224	0,9857907	lm.nb
LILRB4	-0,008291	0,4594638	-0,018	-0,908840399	0,892257837	0,98579	0,9857907	lm.nb
CLEC5A	-0,014279	0,7487137	-0,0191	-1,481757672	1,453200095	0,98498	0,9857907	lm.nb
BTK	-0,015874	0,2808461	-0,0565	-0,566332483	0,534584324	0,95552	0,9693639	lm.nb
NFATC3	-0,019771	0,1000191	-0,1977	-0,215808111	0,176266882	0,8454	0,9098997	lm.nb
IKZF2	-0,025945	0,2342042	-0,1108	-0,484985026	0,433095508	0,91295	0,9404131	lm.nb
IRAK4	-0,026237	0,1654615	-0,1586	-0,350541341	0,298067656	0,87568	0,9287544	lm.nb
BAX	-0,027677	0,1182784	-0,234	-0,259502278	0,204149239	0,81749	0,8921405	lm.nb
CX3CR1	-0,033861	0,3660436	-0,0925	-0,751306575	0,683584194	0,92726	0,9446146	lm.nb
B2M	-0,035401	0,258694	-0,1368	-0,542441786	0,471638808	0,89259	0,9297781	lm.nb

MAPK11	-0,038725	0,2177387	-0,1779	-0,465493304	0,388042566	0,86072	0,9188528	lm.nb
TAGAP	-0,03937	0,3964875	-0,0993	-0,816486002	0,737745081	0,92194	0,9431137	lm.nb
CFH	-0,041208	0,2883254	-0,1429	-0,606325396	0,523910228	0,88786	0,9297781	lm.nb
CD48	-0,045759	0,3324003	-0,1377	-0,697264115	0,605745249	0,89195	0,9297781	lm.nb
BCL10	-0,047215	0,1455323	-0,3244	-0,332458492	0,238028046	0,74916	0,836188	lm.nb
TIRAP	-0,05205	0,1040272	-0,5004	-0,255943387	0,151843209	0,62257	0,7678569	loglinear
IRF8	-0,052528	0,351022	-0,1496	-0,740531574	0,635474785	0,88262	0,9297781	lm.nb
IL1R2	-0,052659	0,6261819	-0,0841	-1,279975182	1,174657977	0,93386	0,9493606	lm.nb
CD164	-0,054635	0,1084756	-0,5037	-0,267246638	0,157977585	0,62029	0,7675327	lm.nb
HLA_B	-0,056709	0,2092992	-0,2709	-0,466934952	0,353517733	0,78935	0,8711348	lm.nb
SYK	-0,061785	0,1813016	-0,3408	-0,417136585	0,293565591	0,737	0,8340041	lm.nb
PRKCD	-0,062161	0,2223516	-0,2796	-0,497970251	0,373648004	0,78283	0,8658886	lm.nb
KCNJ2	-0,063675	0,3250289	-0,1959	-0,700731832	0,573381608	0,84676	0,9098997	lm.nb
JAK1	-0,064379	0,0735007	-0,8759	-0,20844003	0,079682723	0,39203	0,6098215	lm.nb
PDCD1LG2	-0,064646	0,3167534	-0,2041	-0,685483028	0,556190432	0,84045	0,9090994	lm.nb
PTGER4	-0,065504	0,1727855	-0,3791	-0,404163362	0,273155762	0,70881	0,8293041	lm.nb
CSF1	-0,065877	0,399902	-0,1647	-0,849684804	0,717931018	0,87089	0,9256795	lm.nb
TNFSF12	-0,070043	0,2416115	-0,2899	-0,543601731	0,403515463	0,77503	0,8591994	lm.nb
TRAF3	-0,070349	0,1652376	-0,4257	-0,394214901	0,253516363	0,67508	0,8107537	lm.nb
CHUK	-0,071098	0,1456754	-0,4881	-0,356621883	0,214425586	0,63109	0,7726812	lm.nb
PTPRC_all	-0,071544	0,2127914	-0,3362	-0,488615494	0,345526978	0,74039	0,8340041	lm.nb
BCAP31	-0,082569	0,0955402	-0,8642	-0,269828198	0,104689353	0,39824	0,613636	lm.nb
S100A9	-0,082711	0,3839751	-0,2154	-0,835302518	0,669879778	0,83174	0,90167	lm.nb
IFNGR1	-0,082849	0,1626849	-0,5093	-0,401711915	0,236013017	0,61643	0,7666279	lm.nb
SLC2A1	-0,08469	0,4303661	-0,1968	-0,92820763	0,758827428	0,84608	0,9098997	lm.nb
CASP8	-0,084708	0,1741988	-0,4863	-0,426137223	0,256721951	0,63234	0,7726812	lm.nb
IL4R	-0,105642	0,2541156	-0,4157	-0,60370903	0,39242418	0,68227	0,812923	lm.nb
ATG5	-0,107045	0,1114268	-0,9607	-0,325442054	0,11135106	0,34878	0,5872783	lm.nb
IL13RA1	-0,109072	0,1652206	-0,6602	-0,43290437	0,214760444	0,51708	0,7108711	lm.nb
TRAF6	-0,114307	0,1081103	-1,0573	-0,326203579	0,097588699	0,30362	0,5469679	lm.nb
S1PR1	-0,115578	0,2098725	-0,5507	-0,526927754	0,295772464	0,58825	0,7485054	lm.nb
IFNAR1	-0,118045	0,1851751	-0,6375	-0,480988159	0,244898331	0,53142	0,7193277	lm.nb
AHR	-0,121812	0,1275347	-0,9551	-0,371780139	0,128155796	0,35151	0,5878452	lm.nb
CXCL12	-0,127664	0,3557427	-0,3589	-0,824919924	0,569591279	0,72365	0,8340041	lm.nb
CD46	-0,127679	0,1323383	-0,9648	-0,387061824	0,131704362	0,34677	0,5859217	lm.nb
CASP2	-0,129809	0,1563435	-0,8303	-0,436242388	0,176624124	0,41669	0,6282415	lm.nb
ENTPD1	-0,134105	0,2387119	-0,5618	-0,601980282	0,333770387	0,58083	0,7469982	lm.nb
EGR1	-0,136711	0,418478	-0,3267	-0,956927497	0,683506265	0,74748	0,836188	lm.nb
MR1	-0,137504	0,2790518	-0,4928	-0,68444514	0,409438093	0,62783	0,7710237	lm.nb
CD244	-0,141869	0,3638292	-0,3899	-0,854973942	0,571236509	0,70092	0,8275963	lm.nb
BLNK	-0,144784	0,4459792	-0,3246	-1,018903054	0,729335555	0,749	0,836188	lm.nb
IL1R1	-0,148632	0,1676359	-0,8866	-0,477198724	0,179934152	0,38636	0,6087336	lm.nb

CSF2RB	-0,153551	0,408317	-0,3761	-0,953852174	0,64675042	0,71104	0,8293041	lm.nb
CD4	-0,156336	0,390692	-0,4002	-0,922092177	0,609420304	0,69351	0,8228064	lm.nb
PTK2	-0,158617	0,1471313	-1,0781	-0,446994495	0,129760136	0,2945	0,5377902	lm.nb
RUNX1	-0,158977	0,2308546	-0,6886	-0,611452185	0,293497862	0,49937	0,6951445	lm.nb
BCL2	-0,162595	0,1652152	-0,9841	-0,486416311	0,161227301	0,33741	0,5775541	lm.nb
SELL	-0,165756	0,3979768	-0,4165	-0,945790311	0,614278597	0,68171	0,812923	lm.nb
C1R	-0,172375	0,2723945	-0,6328	-0,706268273	0,361518299	0,5344	0,7204479	lm.nb
IL2RG	-0,173198	0,297	-0,5832	-0,755317899	0,408922003	0,56665	0,7349765	lm.nb
TYK2	-0,173995	0,1116173	-1,5589	-0,392765165	0,044774805	0,13553	0,327631	lm.nb
CSF3R	-0,174023	0,4198232	-0,4145	-0,996876766	0,648830295	0,68314	0,812923	lm.nb
CEBPB	-0,174702	0,2419499	-0,7221	-0,648924235	0,299519315	0,47905	0,6772254	lm.nb
SIGIRR	-0,176725	0,2556331	-0,6913	-0,677765649	0,324315982	0,49772	0,6948281	lm.nb
ATG7	-0,176795	0,2249219	-0,786	-0,617642162	0,264051626	0,44154	0,6428265	lm.nb
RIPK1	-0,183874	0,132799	-1,3846	-0,444160551	0,076411698	0,18222	0,3926619	lm.nb
ICAM2	-0,184186	0,2033037	-0,906	-0,582660895	0,214289762	0,3763	0,6039118	lm.nb
SKI	-0,191665	0,0988324	-1,9393	-0,385376375	0,002046485	0,06746	0,2118975	lm.nb
EDNRB	-0,194585	0,37648	-0,5169	-0,93248549	0,543316252	0,61122	0,7620852	lm.nb
ITGAL	-0,199155	0,2805563	-0,7099	-0,749045441	0,350735271	0,48641	0,6816587	lm.nb
FCGR2B	-0,202756	0,3077593	-0,6588	-0,805964265	0,400452088	0,51792	0,7108711	lm.nb
JAK3	-0,208727	0,2543107	-0,8208	-0,707176089	0,289722048	0,42196	0,6305842	lm.nb
NFATC1	-0,210107	0,1420163	-1,4795	-0,488459238	0,068244692	0,15541	0,3548276	lm.nb
ITGAE	-0,214002	0,1378853	-1,552	-0,484256774	0,056253452	0,13715	0,3288538	lm.nb
HFE	-0,215506	0,4766691	-0,4521	-1,149777431	0,71876536	0,65631	0,7960196	lm.nb
CD247	-0,229989	0,4274047	-0,5381	-1,067702372	0,607724175	0,59675	0,7528716	lm.nb
IL16	-0,24329	0,2155108	-1,1289	-0,665691181	0,17911117	0,27299	0,5184749	lm.nb
LY96	-0,243663	0,2635898	-0,9244	-0,760299428	0,272972544	0,36687	0,6012222	lm.nb
BCL6	-0,243825	0,3105872	-0,785	-0,852575524	0,364926206	0,44211	0,6428265	lm.nb
CLEC4A	-0,245287	0,4660892	-0,5263	-1,158822241	0,66824754	0,60479	0,7559903	lm.nb
APP	-0,245718	0,1208927	-2,0325	-0,482667214	-0,008767819	0,05632	0,1999661	lm.nb
HLA_DQB1	-0,248518	2,0727919	-0,1199	-4,311190528	3,81415387	0,90582	0,9383807	loglinear
MAPK14	-0,249855	0,1261117	-1,9812	-0,497034188	-0,002676324	0,06223	0,2046376	lm.nb
CD58	-0,253764	0,1926493	-1,3172	-0,631356741	0,123828389	0,20343	0,4223669	lm.nb
CFLAR_iso1	-0,255707	0,1555706	-1,6437	-0,560625572	0,049211217	0,11669	0,3033855	lm.nb
TLR9	-0,259387	0,3185401	-0,8143	-0,88372518	0,364951922	0,42556	0,6310126	lm.nb
HLA_DQA1	-0,260898	2,3835597	-0,1095	-4,932674817	4,41087911	0,91399	0,9404131	loglinear
STAT5B	-0,262188	0,1057213	-2,48	-0,469401511	-0,054974201	0,02268	0,1133788	lm.nb
HLA_DPB1	-0,26452	0,4372904	-0,6049	-1,121609503	0,592568773	0,5524	0,7276196	lm.nb
DUSP4	-0,271451	0,27192	-0,9983	-0,804414035	0,26151233	0,33069	0,5766497	lm.nb
CD40LG	-0,271989	0,4943705	-0,5502	-1,240955116	0,696977404	0,58861	0,7485054	lm.nb
CTNNB1	-0,27565	0,1070556	-2,5748	-0,48547889	-0,065821067	0,01855	0,1010164	lm.nb
NLRP1	-0,277508	0,1361071	-2,0389	-0,544277557	-0,010737676	0,05562	0,1989329	lm.nb
TLR2	-0,278386	0,4488763	-0,6202	-1,158183816	0,601411207	0,5425	0,7204479	lm.nb

LAG3	-0,279336	0,723632	-0,386	-1,697654905	1,138982469	0,70377	0,8289008	lm.nb
IL11RA	-0,282934	0,2863669	-0,988	-0,844212996	0,278345069	0,33556	0,577137	lm.nb
CXCR3	-0,29708	0,4835197	-0,6144	-1,244778517	0,650618843	0,54623	0,7214379	lm.nb
TNFAIP6	-0,301396	0,4426222	-0,6809	-1,168935354	0,56614381	0,50413	0,6958404	lm.nb
SOCS3	-0,304729	0,429767	-0,7091	-1,147072861	0,537613895	0,4869	0,6816587	lm.nb
NCF4	-0,304889	0,2183998	-1,396	-0,7329526	0,123174757	0,17881	0,3926619	lm.nb
LTF	-0,311478	0,7000438	-0,4449	-1,68356339	1,060608131	0,66139	0,7982297	lm.nb
MAPKAPK2	-0,31755	0,1423502	-2,2308	-0,596556819	-0,038544041	0,03795	0,1524062	lm.nb
ITGAM	-0,320831	0,3795965	-0,8452	-1,064840492	0,423177742	0,40852	0,6236008	lm.nb
C2	-0,323385	0,408016	-0,7926	-1,123096931	0,476325979	0,43781	0,6422926	lm.nb
ADA	-0,325408	0,2783851	-1,1689	-0,871042366	0,220227228	0,2569	0,4995204	lm.nb
IL1RL1	-0,326438	0,5023641	-0,6498	-1,311071471	0,658195989	0,5236	0,71179	lm.nb
ICAM3	-0,33263	0,2180052	-1,5258	-0,759920193	0,094660185	0,14354	0,3381395	lm.nb
IL2RB	-0,333358	0,3506779	-0,9506	-1,020686909	0,353970593	0,35374	0,5884707	lm.nb
S100A8	-0,336499	0,4273814	-0,7873	-1,174166024	0,501168888	0,44079	0,6428265	lm.nb
LCP2	-0,336761	0,4104016	-0,8206	-1,141147843	0,467626442	0,42207	0,6305842	lm.nb
FCER1A	-0,345778	0,4807572	-0,7192	-1,288062345	0,596506023	0,48075	0,6772254	lm.nb
NFKBIA	-0,347347	0,2923328	-1,1882	-0,920319055	0,225625624	0,2494	0,4918289	lm.nb
FCGR2A	-0,351497	0,3632283	-0,9677	-1,063424874	0,36043015	0,34535	0,5855416	lm.nb
CD276	-0,355624	0,1789972	-1,9868	-0,706458476	-0,004789552	0,06156	0,2039645	lm.nb
CCL5	-0,356567	0,4263459	-0,8363	-1,192204589	0,479071235	0,41336	0,6280982	lm.nb
MARCO	-0,358709	0,5805796	-0,6178	-1,496645131	0,779226762	0,54401	0,7204479	lm.nb
FOXP3	-0,359091	0,4618719	-0,7775	-1,264359613	0,546178323	0,44646	0,6446022	lm.nb
ABL1	-0,360678	0,131237	-2,7483	-0,617902366	-0,103453422	0,01278	0,0824111	lm.nb
CRADD	-0,361347	0,1526244	-2,3676	-0,6604905	-0,062202738	0,02867	0,1288921	lm.nb
KLRK1	-0,374184	0,5769683	-0,6485	-1,505042178	0,756673514	0,5244	0,71179	lm.nb
NFKBIZ	-0,380104	0,2868964	-1,3249	-0,942420881	0,182212841	0,20092	0,4189487	lm.nb
ZAP70	-0,395033	0,4381203	-0,9017	-1,253748276	0,463683196	0,37853	0,6039118	lm.nb
ITGA4	-0,395597	0,2701369	-1,4644	-0,925065423	0,133871046	0,15943	0,3599953	lm.nb
MAP4K2	-0,402965	0,1746372	-2,3074	-0,745253627	-0,060675707	0,03245	0,1399531	lm.nb
TRAF1	-0,403836	0,3741772	-1,0793	-1,13722331	0,329551384	0,29398	0,5377902	lm.nb
ABCB1	-0,407955	0,2015101	-2,0245	-0,802915243	-0,012995559	0,05721	0,2003247	lm.nb
IL6	-0,408538	0,7716089	-0,5295	-1,920891493	1,103815278	0,60262	0,7559903	lm.nb
TGFBI	-0,408917	0,2512803	-1,6273	-0,901426708	0,083592247	0,12014	0,3082026	lm.nb
TNFRSF1B	-0,410329	0,3224339	-1,2726	-1,042299648	0,221641397	0,21851	0,4498812	lm.nb
CD99	-0,413467	0,2680069	-1,5427	-0,938760277	0,111826679	0,13938	0,3299426	lm.nb
TGFBR1	-0,423731	0,1897126	-2,2335	-0,79556753	-0,051894311	0,03773	0,1524062	lm.nb
LITAF	-0,431867	0,3422058	-1,262	-1,102590045	0,238856598	0,22222	0,4555937	lm.nb
AnnexinA1	-0,432874	0,3129997	-1,383	-1,046353109	0,180605692	0,18271	0,3926619	lm.nb
GPR183	-0,433835	0,257036	-1,6878	-0,937625585	0,069955396	0,10779	0,2914964	lm.nb
CD96	-0,436517	0,4555334	-0,9583	-1,329362612	0,456328159	0,34997	0,5872783	lm.nb
KIT	-0,453072	0,2904665	-1,5598	-1,022386313	0,116242306	0,13531	0,327631	lm.nb

LIF	-0,461641	0,6231609	-0,7408	-1,683036225	0,759754319	0,46787	0,6664409	lm.nb
FYN	-0,467311	0,2040493	-2,2902	-0,86724726	-0,067373816	0,03362	0,1420225	lm.nb
IKZF1	-0,470679	0,2814039	-1,6726	-1,022231019	0,080872299	0,11079	0,2934445	lm.nb
BCL2L11	-0,474945	0,251728	-1,8867	-0,968331564	0,018442328	0,07457	0,2269612	lm.nb
FLG1	-0,480267	0,7700467	-0,6237	-1,989558563	1,029024564	0,54025	0,7204479	lm.nb
CXCR2	-0,480371	0,42371	-1,1337	-1,310842943	0,350100235	0,27101	0,5174621	lm.nb
NFATC2	-0,503681	0,2657627	-1,8952	-1,024576148	0,017213659	0,07338	0,2247286	lm.nb
IL21R	-0,507361	0,4925061	-1,0302	-1,472673055	0,457950976	0,31587	0,5607842	lm.nb
IL1RN	-0,510092	0,5877775	-0,8678	-1,662135647	0,641951998	0,39632	0,613636	lm.nb
PLAUR	-0,520175	0,4833769	-1,0761	-1,467594288	0,427243293	0,29535	0,5377902	lm.nb
CD82	-0,520433	0,2168828	-2,3996	-0,945523004	-0,095342378	0,02683	0,1240125	lm.nb
CD5	-0,529082	0,5181723	-1,0211	-1,544699737	0,486535636	0,32005	0,5641249	lm.nb
CD3E	-0,534356	0,3908547	-1,3671	-1,300430715	0,231719614	0,18754	0,4012815	lm.nb
TNFRSF4	-0,534394	0,4766864	-1,1211	-1,468698923	0,39991157	0,27623	0,520592	lm.nb
TNF	-0,535672	0,3969503	-1,3495	-1,313694725	0,242350327	0,19305	0,4111532	lm.nb
TNFAIP3	-0,545455	0,4111183	-1,3268	-1,351246938	0,260336947	0,20031	0,4189487	lm.nb
CTLA4_all	-0,549709	0,5118987	-1,0739	-1,553030963	0,453612099	0,29633	0,5377902	lm.nb
TFRC	-0,550544	0,2338854	-2,3539	-1,008959207	-0,092128429	0,02949	0,1301938	lm.nb
PTPN22	-0,557128	0,2261721	-2,4633	-1,000425516	-0,11383107	0,02349	0,1146492	lm.nb
CFLAR	-0,566453	0,1706895	-3,3186	-0,901004175	-0,231901191	0,00361	0,0487888	lm.nb
SLAMF1	-0,568138	0,4634623	-1,2259	-1,476523958	0,340248202	0,23523	0,4762936	lm.nb
CCR7	-0,568877	0,5462572	-1,0414	-1,639541333	0,501786977	0,31076	0,555733	lm.nb
CDH5	-0,571589	0,2049347	-2,7891	-0,973260893	-0,169917	0,0117	0,0796067	lm.nb
CCL22	-0,57778	0,711877	-0,8116	-1,973058641	0,817499054	0,42706	0,6310126	lm.nb
CD14	-0,577985	0,3429448	-1,6854	-1,250157114	0,094186543	0,10827	0,2914964	lm.nb
GZMK	-0,578875	0,4877561	-1,1868	-1,534877221	0,37712688	0,24993	0,4918289	lm.nb
CD2	-0,591745	0,4738239	-1,2489	-1,520439984	0,336949794	0,22688	0,4632152	lm.nb
CTLA4_TM	-0,595323	0,5455897	-1,0912	-1,66467882	0,474032795	0,28885	0,5377902	lm.nb
IL18R1	-0,596548	0,3101293	-1,9235	-1,204401665	0,011305255	0,06953	0,2162868	lm.nb
CR1	-0,597192	0,4169423	-1,4323	-1,414398486	0,22001521	0,1683	0,3748544	lm.nb
CXCR6	-0,600002	0,6570907	-0,9131	-1,887900244	0,68789549	0,37262	0,6039118	lm.nb
TCF4	-0,604206	0,2321666	-2,6025	-1,05925216	-0,14915901	0,01749	0,099998	lm.nb
IRAK2	-0,616346	0,5843995	-1,0547	-1,761768818	0,529077144	0,30481	0,5470877	lm.nb
CASP3	-0,621591	0,1560302	-3,9838	-0,927410559	-0,315772287	0,0008	0,0248921	lm.nb
NFIL3	-0,623438	0,2477862	-2,516	-1,109098861	-0,13777693	0,02102	0,1092503	lm.nb
IL36Ra	-0,627097	0,3727528	-1,6823	-1,35769268	0,103498455	0,10886	0,2914964	lm.nb
LCK	-0,645298	0,4098868	-1,5743	-1,448676234	0,158079906	0,13192	0,324547	lm.nb
IL1RAP	-0,649458	0,4289057	-1,5142	-1,490112749	0,191197413	0,14643	0,3433011	lm.nb
LTA	-0,664468	0,4010342	-1,6569	-1,450495421	0,121558821	0,11396	0,3002262	lm.nb
ICOS	-0,670627	0,5588144	-1,2001	-1,765903308	0,424649122	0,24486	0,4867629	lm.nb
TCF7	-0,676474	0,3471313	-1,9488	-1,356851756	0,003903005	0,06625	0,2094251	lm.nb
THY1	-0,686304	0,3570915	-1,9219	-1,386203715	0,013594977	0,06974	0,2162868	lm.nb

PDGFB	-0,687768	0,1829312	-3,7597	-1,046313515	-0,329223292	0,00133	0,0342038	lm.nb
CD34	-0,691605	0,4038338	-1,7126	-1,483119313	0,099909191	0,10306	0,2869254	lm.nb
CCL3	-0,692638	0,759407	-0,9121	-2,181075895	0,795799481	0,37316	0,6039118	lm.nb
MAP4K1	-0,713831	0,3805888	-1,8756	-1,459784776	0,032123177	0,07616	0,2284262	lm.nb
MAP4K4	-0,735478	0,1769667	-4,156	-1,082332611	-0,388623136	0,00054	0,0219089	lm.nb
PRDM1	-0,736976	0,3012544	-2,4464	-1,327434083	-0,146516991	0,02433	0,1146492	lm.nb
C3	-0,739731	0,4345938	-1,7021	-1,591534533	0,112072967	0,10504	0,2907852	lm.nb
IRAK3	-0,751062	0,2691965	-2,79	-1,278687263	-0,223437024	0,01167	0,0796067	lm.nb
TRAF5	-0,756273	0,2891107	-2,6159	-1,322929964	-0,189615973	0,017	0,0995332	lm.nb
POU2F2	-0,759634	0,2484925	-3,057	-1,246678914	-0,272588138	0,00649	0,0567676	lm.nb
PLAU	-0,778638	0,3775603	-2,0623	-1,518655949	-0,038619565	0,05312	0,1940277	lm.nb
TNFRSF10C	-0,781535	0,4874807	-1,6032	-1,736996858	0,173927354	0,12538	0,315616	lm.nb
PECAM1	-0,783138	0,2430455	-3,2222	-1,259507564	-0,30676917	0,00449	0,0499501	lm.nb
S100A7	-0,796703	0,4733287	-1,6832	-1,724427768	0,131020807	0,1087	0,2914964	lm.nb
CFI	-0,804198	0,2293137	-3,507	-1,253652862	-0,354743216	0,00236	0,0412599	lm.nb
ITGA5	-0,807143	0,2163147	-3,7313	-1,23112012	-0,383166564	0,00141	0,034666	lm.nb
IL18RAP	-0,808302	0,6046663	-1,3368	-1,993448058	0,37684367	0,19708	0,4144679	lm.nb
CEACAM6	-0,818061	0,7615193	-1,0742	-2,310638453	0,674517069	0,29617	0,5377902	lm.nb
CD24	-0,824854	0,4317818	-1,9103	-1,67114577	0,021438768	0,0713	0,2197291	lm.nb
IL36g	-0,83125	0,5335345	-1,558	-1,876977719	0,214477583	0,13573	0,327631	lm.nb
TAL1	-0,840062	0,3426945	-2,4513	-1,511743489	-0,168381023	0,02408	0,1146492	lm.nb
TNFRSF8	-0,848507	0,3002369	-2,8261	-1,436971553	-0,260043095	0,01079	0,0786385	lm.nb
CD28	-0,862562	0,2718435	-3,173	-1,395375647	-0,329749056	0,00501	0,0511233	lm.nb
VEGFA	-0,887351	0,4504748	-1,9698	-1,770281873	-0,004420752	0,06361	0,2060017	lm.nb
TNFRSF9	-0,909121	0,4308321	-2,1102	-1,753551581	-0,064689749	0,04833	0,1821744	lm.nb
SPP1	-0,920123	0,8175948	-1,1254	-2,522608635	0,682363046	0,27443	0,5192005	lm.nb
IVL	-0,922661	0,4356801	-2,1177	-1,776594169	-0,068728136	0,04761	0,1808375	lm.nb
ITGB1	-0,938482	0,1216288	-7,716	-1,176874569	-0,700089723	2,86E-07	4,66E-05	lm.nb
CD3D	-0,941263	0,4342698	-2,1675	-1,792432104	-0,090094677	0,04311	0,1689953	lm.nb
TIGIT	-0,989737	0,4650225	-2,1284	-1,90118072	-0,078292349	0,04661	0,1784434	lm.nb
C1S	-0,992812	0,3116748	-3,1854	-1,603694656	-0,381929363	0,00487	0,0507814	lm.nb
C4AB	-0,998995	0,4839845	-2,0641	-1,947604443	-0,050385132	0,05294	0,1940277	lm.nb
ETS1	-1,001032	0,1653086	-6,0555	-1,325037224	-0,677027593	7,98E-06	0,0007823	lm.nb
DPP4	-1,005697	0,3626915	-2,7729	-1,716571919	-0,294821153	0,01212	0,0802424	lm.nb
ZEB1	-1,020944	0,1988271	-5,1348	-1,410645197	-0,631242832	5,89E-05	0,0048086	lm.nb
PDGFRB	-1,026458	0,2327268	-4,4106	-1,48260247	-0,570313356	0,0003	0,0163557	lm.nb
C7	-1,026945	0,6830244	-1,5035	-2,365673035	0,311782738	0,14914	0,3447197	lm.nb
BTLA	-1,032721	0,3237852	-3,1895	-1,667339937	-0,398101877	0,00483	0,0507814	lm.nb
NLRP3	-1,040636	0,5147051	-2,0218	-2,04945823	-0,031814069	0,05751	0,2003247	lm.nb
HLA_DOB	-1,041288	0,4285708	-2,4297	-1,881287022	-0,201289395	0,0252	0,1175877	lm.nb
CXCR1	-1,042326	0,6379333	-1,6339	-2,292674906	0,208023777	0,11874	0,306221	lm.nb
CD6	-1,074078	0,5704587	-1,8828	-2,19217728	0,044020993	0,07513	0,2272355	lm.nb

XBP1	-1,109112	0,3351108	-3,3097	-1,765929286	-0,452294865	0,00368	0,0487888	lm.nb
MME	-1,120537	0,4053173	-2,7646	-1,914959378	-0,326115439	0,01234	0,0806088	lm.nb
CD45RA	-1,154734	0,4417293	-2,6141	-2,020523371	-0,288944418	0,01706	0,0995332	lm.nb
CXCR4	-1,228198	0,4353815	-2,821	-2,081545423	-0,374850117	0,01091	0,0786385	lm.nb
IKZF3	-1,266566	0,5759284	-2,1992	-2,39538579	-0,137746403	0,04045	0,1611363	lm.nb
IL2RA	-1,319292	0,5749444	-2,2946	-2,446183431	-0,192401263	0,03332	0,1419558	lm.nb
ARG1	-1,334013	0,6478457	-2,0592	-2,603790913	-0,064235709	0,05345	0,1940277	lm.nb
CD69	-1,365006	0,5280817	-2,5848	-2,400046455	-0,329966126	0,01816	0,099998	lm.nb
TNFSF15	-1,396818	0,7749511	-1,8025	-2,91572195	0,122086355	0,08736	0,2518026	lm.nb
IL1B	-1,408765	0,8125242	-1,7338	-3,001312196	0,183782702	0,09915	0,2792114	lm.nb
IL7R	-1,442879	0,4343649	-3,3218	-2,294234271	-0,591523769	0,00358	0,0487888	lm.nb
DEFB4A	-1,442917	1,5963873	-0,9039	-4,571836436	1,686001662	0,37739	0,6039118	loglinear
DEFB103B	-1,480299	0,8174303	-1,8109	-3,082462744	0,121864011	0,086	0,2493358	lm.nb
PTGS2	-1,566692	0,6754495	-2,3195	-2,89057259	-0,242810432	0,03166	0,1385175	lm.nb
CD22	-1,582288	0,5451891	-2,9023	-2,650858993	-0,51371774	0,00913	0,069924	lm.nb
CXCL2	-1,66831	0,6190329	-2,695	-2,881614665	-0,455005844	0,01434	0,0889585	lm.nb
IRF4	-1,677463	0,4171082	-4,0217	-2,494995501	-0,859931261	0,00073	0,0248921	lm.nb
KLRB1	-1,679762	0,5132384	-3,2729	-2,685708804	-0,673814343	0,004	0,0499501	lm.nb
LEF1	-1,748033	0,3650816	-4,7881	-2,463593287	-1,032473596	0,00013	0,0089498	lm.nb
CCL4	-1,914328	0,7400299	-2,5868	-3,364786724	-0,463869632	0,01809	0,099998	lm.nb
CD27	-2,01628	0,5783839	-3,4861	-3,149912925	-0,882648041	0,00247	0,0417737	lm.nb
CCL18	-2,039957	0,5553837	-3,6731	-3,1285085	-0,951404567	0,00162	0,0359923	lm.nb
CXCL1	-2,106281	0,5882432	-3,5806	-3,259237902	-0,953324583	0,00199	0,0398714	lm.nb
PPBP	-2,137679	1,0746525	-1,9892	-4,243997917	-0,031359988	0,06127	0,2039645	loglinear
IL1A	-2,148908	0,8509452	-2,5253	-3,816760931	-0,481055643	0,02061	0,1085834	lm.nb
CD79B	-2,46437	0,5239132	-4,7038	-3,491239332	-1,437499751	0,00015	0,0094692	lm.nb
IL8	-2,624514	0,89051	-2,9472	-4,369913348	-0,879113958	0,00827	0,067561	lm.nb
CCL20	-2,645181	0,7829249	-3,3786	-4,179713474	-1,110647973	0,00315	0,0454474	lm.nb
FN1	-2,796818	0,3321261	-8,421	-3,447785388	-2,145850929	7,76E-08	3,80E-05	lm.nb
CD19	-3,24621	0,4667863	-6,9544	-4,161110938	-2,331308629	1,26E-06	0,0001539	lm.nb
CXCL13	-5,402207	0,6926567	-7,7993	-6,759813811	-4,044599689	2,44E-07	4,66E-05	lm.nb
