# Supplementary material for: Type I and II Interferon Signalling Characterizes the Transcriptional Landscape of Sweet Syndrome
Source: Exp Dermatol. 2026 Jul 6;35(7):e70323. doi: 10.1111/exd.70323 (PMC13338580; doi:10.1111/exd.70323)
Supplement: Supplementary file 3 — Table S3: Clinical and demographics features of patients with Sweet Syndrome. Table detailing demographic data, clinical features, disease subtype, associated conditions, and treatment information for patients included in the Sweet syndrome cohort. [file EXD-35-e70323-s002.docx]

**Supplementary Table 3. Clinical and demographics features of patients with Sweet Syndrome.**

| **N.** | **Sex** | **Age (years)** | **Comorbidities** | **Histopathological features** | **Target lesion location** | **Treatments** | **Outcome** |
| --- | --- | --- | --- | --- | --- | --- | --- |
| Sweet_01 | M | 69 | Arthrosis | Acanthosis with diffuse spongiosis. Neutrophil-filled intraepidermal vesicles. In the upper dermis, oedema and dense perivascular and interstitial neutrophilic infiltrate with nuclear dust | Trunk | Prednisone | Complete resolution |
| Sweet_03 | M | 60 | None | Acanthosis with diffuse spongiosis. Neutrophil-filled intraepidermal vesicles. In the upper dermis, oedema and dense perivascular and interstitial neutrophilic infiltrate with nuclear dust | Trunk | Prednisone | Complete resolution |
| Sweet_09 | F | 50 | Hypertension | Acanthosis with diffuse spongiosis. Neutrophil-filled intraepidermal vesicles. In the upper dermis, oedema and dense perivascular and interstitial neutrophilic infiltrate with nuclear dust | Upper limbs | Prednisone | Complete resolution |
| Sweet_10 | M | 64 | Hypertension | Acanthosis with diffuse spongiosis. Neutrophil-filled intraepidermal vesicles. In the upper dermis, oedema and dense perivascular and interstitial neutrophilic infiltrate with nuclear dust | Trunk | Deltacortene | Complete resolution |
| Sweet_11 | F | 69 | Hypertension | Dense dermal perivascular and interstitial neutrophilic infiltrate | Upper limbs | Deltacortene | Complete resolution |
| Sweet_12 | F | 53 | None | Dense dermal perivascular and interstitial neutrophilic infiltrate | Upper limbs | Deltacortene | Complete resolution |
| Sweet_13 | M | 68 | Arterial hypertension, sleep apnea, iron deficiency, vitamin B12 deficiency, type 2 diabetes mellitus, type C gastritis | Acanthosis with diffuse spongiosis. In the upper dermis, oedema and dense perivascular and interstitial neutrophilic infiltrate with nuclear dust | Limbs | Topical steroids | Complete resolution |
| Sweet_14 | F | 75 | Breast cancer (status post bilateral mastectomy), status post spinal canal stenosis | Dense dermal perivascular and interstitial neutrophilic infiltrate | Limbs | Prednisone | Complete resolution |
| Sweet_15 | F | 49 | Hypertension | Dense dermal perivascular and interstitial neutrophilic infiltrate | Limbs | Prednisone | Complete resolution |
| Sweet_16 | F | 81 | Hypertension, breast cancer | Dense dermal perivascular and interstitial neutrophilic infiltrate | Limbs | Topical steroids | Complete resolution |
| Sweet_17 | F | 52 | Colitis ulcerosa, atypical pneumonia | Dense dermal perivascular and interstitial neutrophilic infiltrate | Trunk | Tofacitinib | Complete resolution |
