# Supplementary material for: Type I and II Interferon Signalling Characterizes the Transcriptional Landscape of Sweet Syndrome
Source: Exp Dermatol. 2026 Jul 6;35(7):e70323. doi: 10.1111/exd.70323 (PMC13338580; doi:10.1111/exd.70323)
Supplement: Supplementary file 4 — Figure S1: Heatmap and PCA of gene expression profiles from skin samples of SS, PG and healthy controls. (a) Unsupervised clustering based on gene expression profile showed partial overlap, but overall segregation between the SS and PG samples; (b) The PCA plot similarly demonstrated partial convergence with preserved separation, supporting molecular relatedness between SS and PG while highlighting distinct underlying programmes. Figure S2: GSEA from preidentifed Cluster 2 (Sweet‐dominant) vs. Cluster 1 + 3 (combined SS/PG). (a) GSEA revealed that Type I and Type II Interferon Signalling are the only two pathways reaching FDR significance in Cluster 2 relative to the other clusters. (b) Cell type deconvolution revealed a consistent enrichment of different cell populations (Th1, CD8+ T cells, etc.), including DCs, in Cluster 2 vs. Cluster 1 + 3. This figure was generated with the assistance of Claude (claude‐sonnet‐4‐6, Anthropic, 2026). [file EXD-35-e70323-s001.docx]

**Type I and II interferon signaling characterizes the transcriptional landscape of Sweet syndrome**

Laura Calabrese^1,2,3*^, Chiara Moltrasio^4*^, Maurizio Romagnuolo^1,4*^, Pia-Charlotte Stadler^3^, Zeno Fiocco^3^, Matthias Neulinger-Muñoz^3^, Rui Aoki^3^, Martina D’Onghia^1^, Pietro Rubegni^1^, Katrin Kerl^5^, Takashi K. Satoh^6,3^, Angelo Valerio Marzano^4,7^, Lars E. French^6^.

^1^ Dermatology Unit, Department of Medical, Surgical and Neurosciences, Siena University Hospital, Siena - Italy

^2^ Institute of Dermatology, Catholic University of the Sacred Heart, Rome, Italy.

^3^ Department of Dermatology and Allergy, University Hospital LMU, Munich, Germany.

^4^ Dermatology Unit, Fondazione IRCCS Ca’ Granda Ospedale Maggiore Policlinico, Milan, Italy

^5^ Department of Dermatology, Universitätsspital Zürich, Zürich, Switzerland

^6^ Dr Phillip Frost Department of Dermatology and Cutaneous Surgery, Miller School of Medicine, University of Miami, Miami, Florida, USA.

^7^ Department of Pathophysiology and Transplantation, Università degli Studi di Milano, Milan, Italy.

* These authors contributed equally to this work

CORRESPONDING AUTHOR

Laura Calabrese

Dermatology Unit, Department of Medical, Surgical and Neurosciences, Siena University Hospital, Siena - Italy

Email: [laura.calabrese@unisi.it](mailto:laura.calabrese@unisi.it)

**CONTENT**

**Supplementary Tables**

**Supplementary Table 1. Differential gene expression analysis of Sweet Syndrome versus healthy controls (HCs).**

Table listing differentially expressed genes identified by NanoString analysis in skin samples from patients with SS versus HCs, including log₂ fold change, adjusted p values, and false discovery rate–corrected significance. The data demonstrate a robust inflammatory transcriptional signature associated with SS.

**Supplementary Table 2. Sweet syndrome–specific transcriptional signatures in comparison with pyoderma gangrenosum and healthy controls.**

Table summarizing differential gene expression analyses comparing SS with PG and HC skin samples. The table highlights a subset of genes selectively upregulated in SS, including interferon-related genes that distinguish SS from both PG and healthy controls.

**Supplementary Table 3. Clinical and demographics features of patients with Sweet Syndrome.**

Table detailing demographic data, clinical features, disease subtype, associated conditions, and treatment information for patients included in the Sweet syndrome cohort.

**
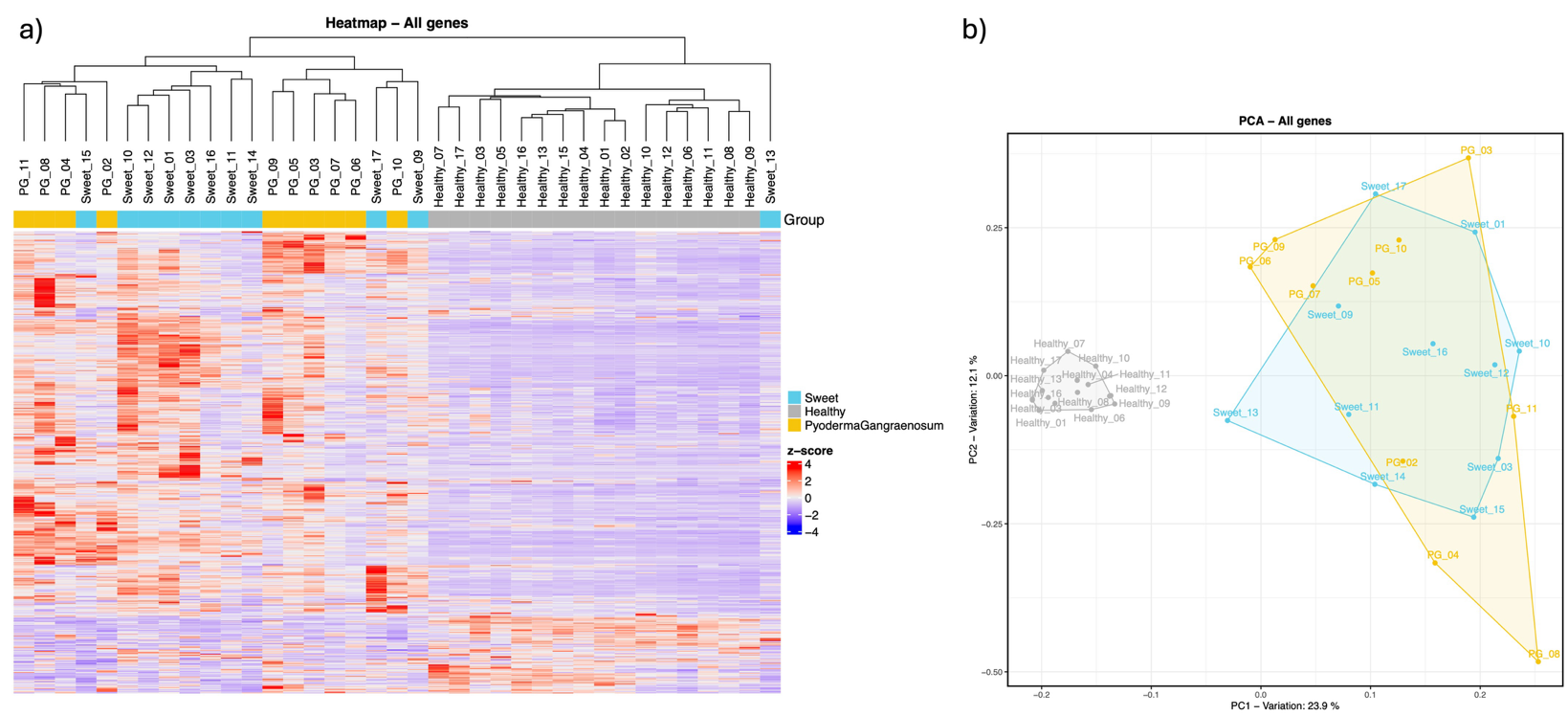
**

**Supplementary Figure 1.** **Heatmap and PCA of gene expression profiles from skin samples of SS, PG and healthy controls.**

a) Unsupervised clustering based on gene expression profile showed partial overlap, but overall segregation between the SS and PG samples; b) The PCA plot similarly demonstrated partial convergence with preserved separation, supporting molecular relatedness between SS and PG while highlighting distinct underlying programs

Legend: PCA, principal component analysis; SS, Sweet syndrome; PG, pyoderma gangrenosum

**
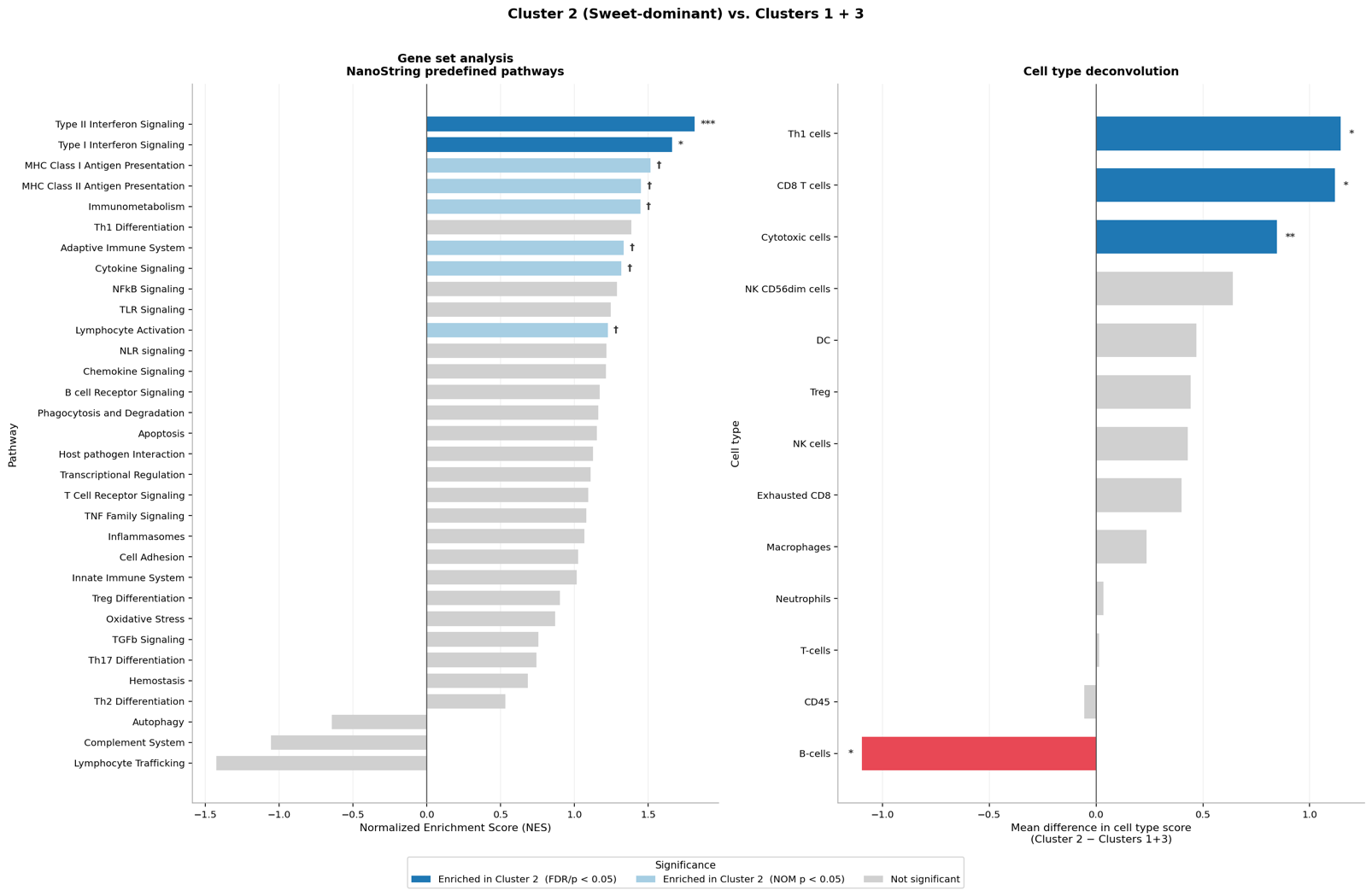
**

**Supplementary figure 2. GSEA from preidentifed Cluster 2 (Sweet-dominant) vs. Cluster 1+3 (combined SS/PG).**

a) GSEA revealed that Type I and Type II Interferon Signaling are the only two pathways reaching FDR significance in Cluster 2 relative to the other clusters. b) Cell type deconvolution revealed a consistent enrichment of different cell populations (Th1, CD8+ T cells, etc.), including DCs, in Cluster 2 vs Cluster 1+3. This figure was generated with the assistance of Claude (claude-sonnet-4-6, Anthropic, 2026).

Legend: GSEA, Gene set Enrichment Analysis; SS, Sweet syndrome; PG, pyoderma gangrenosum; DC, dendritic cells.
